# Induction of HIF-1α by HIV-1 Infection in CD4^+^ T Cells Promotes Viral Replication and Drives Extracellular Vesicle-Mediated Inflammation

**DOI:** 10.1128/mBio.00757-18

**Published:** 2018-09-11

**Authors:** Gabriel Duette, Pehuen Pereyra Gerber, Julia Rubione, Paula S. Perez, Alan L. Landay, Suzanne M. Crowe, Zhaohao Liao, Kenneth W. Witwer, María Pía Holgado, Jimena Salido, Jorge Geffner, Omar Sued, Clovis S. Palmer, Matias Ostrowski

**Affiliations:** a INBIRS, Facultad de Medicina, Buenos Aires, Argentina; b Department of Immunology-Microbiology, Rush University Medical Center, Chicago, Illinois, USA; c Life Sciences Discipline, Burnet Institute, Melbourne, Victoria, Australia; d Department of Infectious Diseases, Monash University, Melbourne, Australia; e Infectious Diseases Department, The Alfred Hospital, Melbourne, Australia; f Department of Molecular and Comparative Pathobiology, The Johns Hopkins University School of Medicine, Baltimore, Maryland, USA; g Fundación Huésped, Buenos Aires, Argentina; h Department of Microbiology and Immunology, Faculty of Medicine, Dentistry, and Health Sciences, The University of Melbourne, Melbourne, Australia; University of Pittsburgh; University of Pittsburgh School of Medicine

**Keywords:** CD4^+^ T lymphocyte, extracellular vesicles, hypoxia-inducible factor 1 alpha, inflammation, human immunodeficiency virus, macrophage

## Abstract

Chronic immune activation and inflammation are hallmarks of HIV-1 infection and a major cause of serious non-AIDS events in HIV-1-infected individuals on antiretroviral treatment (ART). Herein, we show that cytosolic double-stranded DNA (dsDNA) generated in infected CD4^+^ T cells during the HIV-1 replication cycle promotes the mitochondrial reactive oxygen species (ROS)-dependent stabilization of the transcription factor hypoxia-inducible factor 1α (HIF-1α), which in turn, enhances viral replication. Furthermore, we show that induction of HIF-1α promotes the release of extracellular vesicles (EVs). These EVs foster inflammation by inducing the secretion of gamma interferon by bystander CD4^+^ T cells and secretion of interleukin 6 (IL-6) and IL-1β by bystander macrophages through an HIF-1α-dependent pathway. Remarkably, EVs obtained from plasma samples from HIV-1-infected individuals also induced HIF-1α activity and inflammation. Overall, this study demonstrates that HIF-1α plays a crucial role in HIV-1 pathogenesis by promoting viral replication and the release of EVs that orchestrate lymphocyte- and macrophage-mediated inflammatory responses.

## Abstract (precis)

Human immunodeficiency virus type 1 (HIV-1) is a very important global pathogen that preferentially targets CD4^+^ T cells and causes acquired immunodeficiency syndrome (AIDS) if left untreated. Although antiretroviral treatment efficiently suppresses viremia, markers of immune activation and inflammation remain higher in HIV-1-infected patients than in uninfected individuals. The hypoxia-inducible factor 1α (HIF-1α) is a transcription factor that plays a fundamental role in coordinating cellular metabolism and function. Here we show that HIV-1 infection induces HIF-1α activity and that this transcription factor upholds HIV-1 replication. Moreover, we demonstrate that HIF-1α plays a key role in HIV-1-associated inflammation by promoting the release of extracellular vesicles which, in turn, trigger the secretion of inflammatory mediators by noninfected bystander lymphocytes and macrophages. In summary, we identify that the coordinated actions of HIF-1α and extracellular vesicles promote viral replication and inflammation, thus contributing to HIV-1 pathogenesis.

## INTRODUCTION

HIV-1 actively replicates in CD4^+^ T lymphocytes, causing progressive cell loss and leading to the development of AIDS (1). Disruption of the HIV-1 viral cycle by the use of combination antiretroviral therapy (cART) prevents AIDS-related diseases (2). However, the risk of suffering from non-AIDS-related diseases, such as cancer, or from cardiovascular, neurological, kidney, and bone diseases, is higher than in noninfected individuals (3). Chronic T cell activation and inflammation predicts and likely contributes to this excess risk of morbidities (4, 5). Thus, a better understanding of the pathogenesis underlying these immune disorders during HIV-1 infection is of utmost importance.

The hypoxia-inducible factor 1α (HIF-1α) is a transcriptional activator factor that plays a central role in coordinating cellular metabolism and function. The activity of HIF-1α can be triggered by hypoxia. In addition, in many cell types, including immune cells, HIF-1α can also be activated in nonhypoxic conditions. In CD4^+^ T cells, antigen recognition through the T cell receptor (TCR) prompts accumulation of HIF-1α mRNA and protein, particularly in the T helper 17 (Th17) CD4^+^ T cell subset (6–8). In addition, it has been proposed that reactive oxygen species (ROS) production by activated T cells (9) may constitute another mechanism of HIF-1α induction in T helper lymphocytes (10). Irrespective of the activation pathway, accumulation of cytosolic HIF-1α is followed by the nuclear translocation of this transcription factor. Next, by binding to the hypoxia-responsive element (HRE) present in the promoters of numerous genes, HIF-1α induces their transcription. Of note, HIF-1α induces the transcription of genes that participate in glycolysis. This metabolic alteration is, in turn, critical for the effector functions of T cells, particularly the Th1 and Th17 subsets, and for the activity of macrophages (8, 10–12).

Others and our group have previously shown that HIV-1 infection is associated with an increase in aerobic glycolysis of CD4^+^ T cells (13, 14). However, the pathways responsible for the increase in glycolytic activity during HIV-1 infection have not been analyzed. In view of the importance of HIF-1α in the regulation of T cell metabolism and the development of inflammatory responses, herein we studied the modulation of HIF-1α activity and its functional consequences in HIV-1-infected CD4^+^ T cells. We show that HIV-1 induces the hypoxia-independent activation of HIF-1α. Remarkably, HIF-1α activity is triggered not only in productively infected cells but also in bystander (noninfected) cells. Moreover, we demonstrate that the induction in bystander cells occurs in a process mediated by extracellular vesicles (EVs) released by infected cells. EVs comprise a heterogeneous group of membrane-surrounded structures secreted by a wide variety of cells. EVs can mediate intercellular communication by promoting the transfer of antigens, immunomodulatory molecules, lipids, and nucleic acids, which can then exert functions in the recipient cells (15). It has been previously shown that, by inducing the expression of Rab22a, a cellular small GTPase involved in intracellular trafficking, HIF-1α can promote the release of EVs by tumor cells (16). The effects of HIF-1α activation in the function of EVs produced by CD4^+^ T cells have not been previously studied.

Overall, our results show that the induction of HIF-1α activity by mitochondrial reactive oxygen species (mtROS) in HIV-1-infected CD4^+^ T cells promotes viral replication and the release of EVs with proinflammatory activity. Moreover, we show that EVs purified from plasma samples from HIV-1-infected individuals also trigger HIF-1α activity and the release of cytokines by bystander macrophages, despite cART treatment and viral loads below the limit of clinical detection. Thus, by controlling viral replication and the release of EVs, HIF-1α plays a critical role in immune dysfunction and the promotion of an inflammatory response during HIV-1 infection.

## RESULTS

### HIF-1α expression and transcriptional activity are induced upon HIV-1 infection.

**FIG 1:**
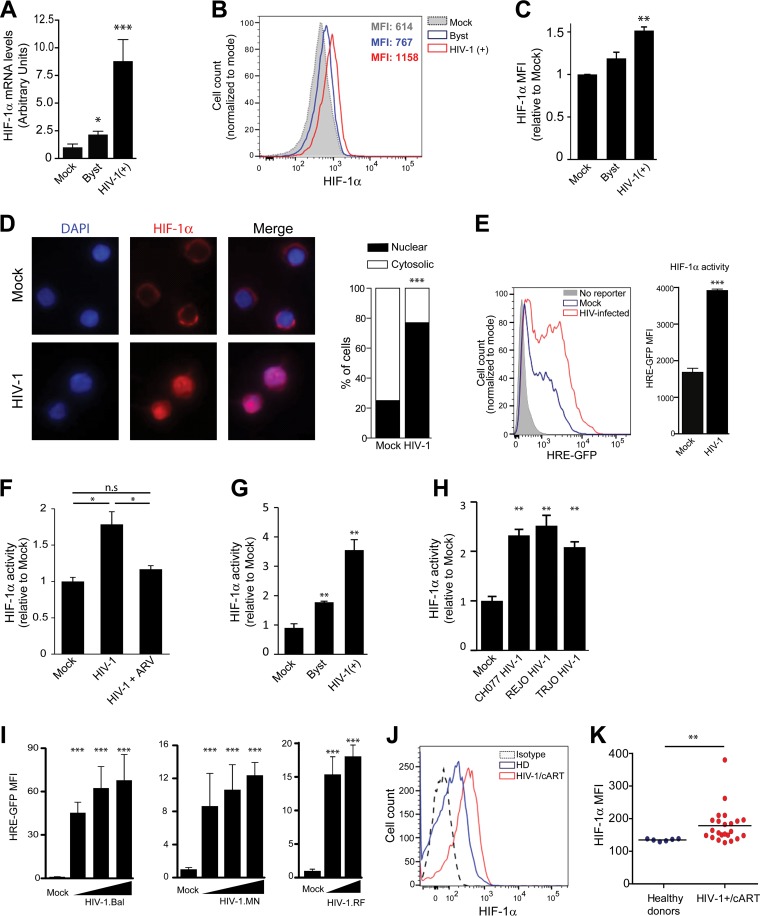
HIV-1 infection increases HIF-1α levels and activity in CD4^+^ T cells. (A to C) CD4^+^ T cells isolated from blood samples from healthy donors were activated through stimulation with anti-CD3/CD28/CD2 antibody-coated beads for 72 h. A total of 10^7^ cells were either mock infected or infected with VSV-G-pseudotyped HIV-1-GFP (200 ng/ml p24). (A) At day 2 postinfection, GFP-positive (GFP+) (productively infected) and GFP-negative cells (bystander [Byst] cells) were sorted by FACS. HIF-1α mRNA levels were determined by qPCR and are expressed as fold change from the value for the control condition (the value for mock-infected cells set at 1). The results of a representative experiment (*n* = 3) performed in triplicate are shown. (B and C) HIF-1α protein levels in mock-infected (filled gray histogram), HIV-1-infected (GFP-positive cells [red histogram]) and bystander (GFP-negative cells [blue histogram]) CD4^+^ T cells were analyzed by intracellular FACS staining. Histograms from a representative experiment (B), and the average fold change (compared to the value for the mock-infected condition) in the mean fluorescent intensity (MFI) obtained with cells from four different donors (C) are shown. (D) Immunofluorescence microscopy of HIV-1-infected CD4^+^ T cells stained with anti-HIF-1α antibodies (red) at day two postinfection. Cell nuclei were stained with 4′,6-diamidino-2-phenylindole (DAPI) (blue). Quantitation of cytosolic versus cytosolic plus nuclear distribution of HIF-1α was evaluated by observers in a blind manner on a per-cell basis in 100 cells of each condition. Data are expressed as a percentage of cells in each category. (E to I) HIF-1α transcriptional activity induced by HIV-1 infection was evaluated by FACS analysis using the Jurkat HRE-GFP cells. (E) Histograms from a representative experiment (left panel), and the MFI of GFP expression (right panel) at 48 h postinfection (p.i.) is shown (*n* = 5). The fluorescence corresponding to mock-infected and HIV-1-infected cells are represented as blue and red histograms, respectively. Cells not expressing the reporter gene are shown as a negative control (filled gray histogram). (F) Cells were pretreated (1 h) with antiretroviral (ARV) drugs (enfurvitide and efavirenz). Cells were then infected, and HIF-1α activity was determined by analyzing the expression of the reporter GFP at 48 h p.i. Pooled data from three independent experiments are shown. (G) Productively infected cells were identified by intracellular staining of p24 antigen. GFP expression in HIV-1-infected Jurkat HRE-GFP cells was analyzed in p24-positive cells (productively infected) or p24-negative cells (bystander cells) versus mock-infected cells. A representative experiment of four independent experiments is shown. (H) Induction of HIF-1α by primary HIV-1 isolates in Jurkat HRE-GFP was analyzed (*n* = 2). (I) Infection of Jurkat HRE-GFP with virus strains of different surface tropism resulted in dose-dependent increase of HIF-1α reporter. The strains were BaL (CCR5-tropic), MN (CXCR5-tropic), and RF (dual-tropic). (J and K) Total HIF-1α levels were determined in CD4^+^ T cells isolated from healthy donors (*n* = 6) and HIV-1-infected donors (*n* = 23) by intracellular FACS staining. A representative histogram (J) and values corresponding to each individual (K) are shown. Statistical significance is indicated as follows: *, *P* < 0.05; **, *P* < 0.005; ***, *P* < 0.0001; n.s., not significant.

HIV-1 infection is associated with an increase in aerobic glycolysis in CD4^+^ T cells, both *in vitro* and in patients (13, 14). However, the stimuli and pathways underlying this virus-induced metabolic alteration, as well as the functional consequences in terms of immune function, are unknown. Taking into consideration that HIF-1α plays a central role in the control of glucose metabolism and in CD4^+^ T cell functionality, we aimed to study whether HIF-1α activity was modulated during HIV-1 infection. CD4^+^ T cells isolated from blood samples from healthy donors were activated. The cells were then either mock infected or infected with NL4-3-IRES-eGFP (HIV-1-GFP), a viral construct that allows the identification of productively infected cells based on green fluorescent protein (GFP) expression. At 48 h postinfection, HIV-1-infected cultures (~10% infected cells) were sorted by fluorescence-activated cell sorting (FACS) based on GFP expression, obtaining two cell populations: productively infected (GFP+) and bystander (GFP−) cells. Analysis of HIF-1α mRNA levels (Fig. 1A) revealed that HIV-1 infection increased the expression of HIF-1α in productively infected cells compared with mock-infected cells. GFP-negative CD4^+^ T cells exhibited a modest but significant increase in HIF-1α expression (Fig. 1A), suggesting that in HIV-1-infected cultures, HIF-1α is induced not only in productively infected cells but also in bystander cells. Similar results were obtained when HIF-1α protein levels were analyzed by FACS in infected CD4^+^ T cells (Fig. 1B and C).

The subcellular localization of HIF-1α was also altered by HIV-1 infection. Whereas in mock-infected cells, HIF-1α exhibited a predominantly cytosolic distribution, in HIV-1-infected cultures, HIF-1α was translocated into the nucleus in the majority of cells (Fig. 1D). The specificity of antibodies used in Fig. 1B and D is shown in Fig. S1A and B, respectively, in the supplemental material. The nuclear translocation of HIF-1α in HIV-1-infected cultures was also observed in Jurkat cells (Fig. S1C).

10.1128/mBio.00757-18.2 FIG S1  (A and B) Specificity of the anti-HIF-1α antibodies used throughout this study. To analyze the specificity of monoclonal mouse IgG1 clone 241812 that was used to detect intracellular HIF-1α levels in Fig. 1B and J, Jurkat cells were treated with CoCl_2_, and intracellular HIF-1α was analyzed by FACS (A). To confirm the specificity of MAb clone 54 used for immunofluorescence in Fig. S1D and S1C, we performed immunoblotting with cells stimulated with CoCl_2_ (B). (C) Immunofluorescence microscopy of HIV-1-infected Jurkat cells stained with anti-HIF-1α antibodies (red) at day 3 postinfection. Cell nuclei were stained with DAPI (blue). Quantitation of cytosolic versus cytosolic plus nuclear distribution of HIF-1α was evaluated in a blind manner by observers on a per-cell basis in 100 cells from each condition. Data are expressed as a percentage of cells in each category (*n* = 2 independent experiments). (D and E) Validation of the HIF-1α reporter cell line (Jurkat HRE-GFP) was performed by stimulation with CoCl_2_ (100 µM) (D). In addition, pharmacological i nhibition of HIF-1α activity with echinomycin (E) (a small-molecule inhibitor of hypoxia-inducible factor 1 DNA-binding activity) (44) abrogated the responsiveness of the reporter cell line to stimulation with CoCl_2_. These results validate the specificity of the reporter cell line. (F and G) CD4^+^ T cells isolated from blood samples from healthy donors were activated and subsequently infected with VSV-G-pseudotyped HIV-1 or mock infected. (F) Cell surface glucose transporter 1 (Glut-1) protein levels in mock-infected (blue histogram) and HIV-1-infected (red histogram) CD4^+^ T cells were analyzed by FACS. Isotype control is shown (filled gray histogram). Histograms from a representative experiment and average MFI (*n* = 5) are shown. (G) Glucose uptake was evaluated by incubating cells for 30 min with the fluorescent glucose analog 6-*N*-(7-nitrobenz-2-oxa-1,3-diazol-4-yl)amino)-6-deoxyglucose (6-NBDG) (14 µM) in culture medium. After the cells were washed, the cells were analyzed by FACS. A representative histogram and average MFI (*n* = 3) are shown. (H) CD4^+^ T cells isolated from blood samples from healthy donors were activated through stimulation with anti-CD3/CD28/CD2 antibody-coated beads. Next, a total of 10^7^ cells were either mock infected or infected with VSV-G-pseudotyped HIV-1-GFP (200 ng/ml p24). On day 3 postinfection, GFP-positive cells (productively infected) and GFP-negative (bystander) cells were sorted by FACS. The mRNA levels of the glycolytic enzyme hexokinase 1 (HK1) were determined by qPCR and are expressed as fold change compared to the value for the control condition (mock = 1). A representative experiment (*n* = 3) performed in triplicate is shown. (I to K) CD4^+^ T cells isolated from blood samples from healthy donors were activated and subsequently infected with VSV-G-pseudotyped HIV-1 or mock infected. (I) Lactate dehydrogenase (LDH) activity was evaluated after cell lysis by measuring the reduction of tetrazolium salt to red formazan by an enzymatic reaction dependent on the amount of LDH present in the cell lysate. Red formazan absorbance was measured at 490 nm using a plate-reading spectrophotometer. A representative experiment (*n* = 4) is shown. (J) The pH of the culture medium from infected and mock-infected cells was quantified as a proxy for glycolysis (acidification due to lactic acid production). (K) The cells were incubated in the presence or absence of echinomycin to quantify the pH of the medium as a proxy for glycolysis (acidification due to lactic acid production). Pooled data from three independent experiments is shown. (L) Comparative relationship between intracellular HIF-1α and cell-surface Glut-1 levels. *, *P* < 0.05; **, *P* < 0.005; ***, *P* < 0.0001; n.s., not significant. Download FIG S1, TIF file, 2.1 MB. Copyright © 2018 Duette et al. 2018 Duette et al. This content is distributed under the terms of the Creative Commons Attribution 4.0 International license.

To analyze the transcriptional activity of HIF-1α, we constructed a reporter Jurkat cell line (Jurkat HRE-GFP), in which the expression of the reporter GFP is under the control of hypoxia-responsive elements (HREs), the DNA regulatory sequences present in the promoter or enhancer regions of HIF-1α target genes (17). Before performing the analysis of HIF-1α activity during HIV-1 infection, we validated the responsiveness and HIF-1α specificity of the reporter cell line (Fig. S1D and E).

We observed that HIV-1 infection enhanced HIF-1α transcriptional activity, as revealed by an increase in the mean fluorescent intensity (MFI) of the GFP reporter expression (Fig. 1E). Pretreatment of cells with a combination of antiretroviral drugs that interrupt the first steps of the viral replication cycle (viral entry [enfurtivide] and reverse transcription [efavirenz]) abrogated the HIV-1-mediated induction of HIF-1α activity (Fig. 1F). These results indicate that HIF-1α activity is actually triggered by HIV-1 infection and rule out the possibility that another factor present in the viral stock could induce the activity of this transcription factor. Next, we decided to analyze whether the increased HIF-1α activity was restricted to productively infected cells or whether it also took place in bystander cells. Paralleling the increase in HIF-1α mRNA (Fig. 1A), the transcriptional activity of HIF-1α was primarily induced in productively infected cells (as revealed by the intracellular detection of the viral antigen p24), but there was also a significant increase in bystander cells (Fig. 1G).

Next, the ability of HIV-1 to induce HIF-1α activity was evaluated by using primary HIV-1 isolates with chemokine (C-X-C motif) receptor 4 (CXCR4) and chemokine (C-C motif) receptor 5 (CCR5) tropisms (Fig. 1H). Likewise, HIV-1 laboratory strains with different tropisms, including HIV-1 strains BaL (CCR5 tropic), MN (CXCR4 tropic), and RF (dual-tropic) also triggered HIF-1α activity in a dose-dependent manner (Fig. 1I). These results show that the induction of HIF-1α by HIV-1 infection is not restricted to viruses with certain tropism or to laboratory strains of HIV-1.

To functionally confirm the increase in HIF-1α activity during HIV-1 infection, we analyzed the glycolytic activity of HIV-1-infected cells. In agreement with previous results (14), we observed that HIV-1 infection promoted an increase in glycolysis, as revealed by an increase in the levels of cell surface glucose transporter 1 (Glut-1) (Fig. S1F), glucose uptake (Fig. S1G), hexokinase 1 (HK1) mRNA (Fig. S1H), lactate dehydrogenase (LDH) activity (Fig. S1I), and extracellular acidification (an indicator of lactic acid production and a proxy for glycolysis; Fig. S1J). Pharmacological inhibition of HIF-1α with echinomycin, a small molecule that inhibits the binding of HIF-1α to its target HRE sequence in the DNA, abolished the acidification of cell culture medium in HIV-1-infected cultures, indicating that this transcription factor plays a critical role in the promotion of glycolytic activity triggered by HIV-1 (Fig. S1K).

Finally, HIF-1α protein levels in CD4^+^ T cells isolated from HIV-1-infected patients were analyzed. A total of 23 HIV-1-infected patients on cART and 6 healthy donors were recruited. Age, sex, and CD4^+^ T cell levels of HIV-1-infected patients and healthy donors are depicted in Table 1. The total cellular levels of HIF-1α were analyzed by intracellular FACS staining. We observed that CD4^+^ T cells from HIV-1-infected patients who were on cART treatment exhibited an increase in cellular HIF-1α levels (Fig. 1J and K). As expected, HIF-1α levels in CD4^+^ T cells from HIV-1-infected patients exhibited a positive correlation with cell surface expression of Glut-1 (Fig. S1L). Altogether, these results show that HIV-1 infection triggers HIF-1α activity *in vitro*, promoting T cell glycolytic activity, and that CD4^+^ T cells from HIV-1-infected patients have higher HIF-1α protein levels than those from HIV-negative healthy individuals.

**TABLE 1:** Clinical characteristics of study groups

Study group and patient	Age (yr)	% CD4^+ ^T cells	CD4^+ ^T cell count^a^	Sex^b^
HIV-negative healthy donors				
10010	27	62.4	ND	M
1009	82	65.7	ND	M
L	55	62	ND	F
T	30	54.4	ND	M
10006	40	53.1	ND	M
10012	65	57.3	ND	M

HIV+ patients on cART				
00010	72	34.7	ND	M
00018	32	37.3	ND	M
00020	51	38.7	ND	M
00021	53	40.3	666	M
00022	63	11.7	296	M
00024	55	20.1	216	M
00027	53	41	728	M
00028	70	52	506	M
00029	60	58	959	M
00030	49	37	917	M
00032	67	36	485	M
00033	66	66	1,377	M
00034	53	24	488	M
00035		49	ND	M
00036		38	1,272	M
00037		47	1,174	M
00038		46	616	M
00039		55	857	M
00040	45	48	360	M
00041		68	951	M
00008 b	44	44.5	ND	M
00016d			470	M
0001G	39	35.5	550	M

a ND, not determined.

b M, male; F, female.

### HIF-1α activity promotes HIV-1 replication.

**FIG 2:**
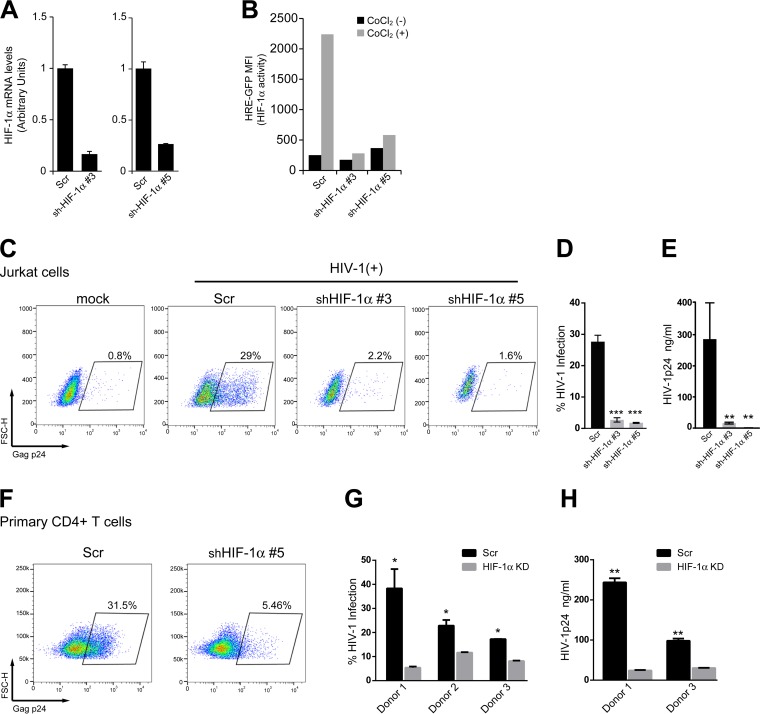
HIF-1α activity promotes HIV-1 replication. (A) The expression of HIF-1α in Jurkat cells was silenced by lentiviral transduction with two specific shRNAs targeting HIF-1α. A scrambled (Scr) shRNA was used as a control. (B) Functional evidence of silencing was obtained by analyzing HIF-1α activity in Jurkat HRE-GFP reporter cells treated with CoCl_2_ [CoCl_2_ (+)]. (C to E) Control or HIF-1α-silenced Jurkat cells were infected with VSV-G-pseudotyped HIV-1 (20 ng/ml p24). On day 3 postinfection, the percentage of infected cells was evaluated by intracellular staining of the viral antigen p24 followed by FACS analysis. A representative dot-plot (C) and the mean plus standard deviation (SD) (error bar) of a representative experiment performed in triplicate (D) are shown. FSC-H, forward scatter height. (E) Viral production was analyzed in cell culture supernatant by ELISA to detect the viral antigen p24. (F to H) Control or HIF-1α-silenced primary CD4^+^ T cells were infected with VSV-G-pseudotyped HIV-1 (20 ng/ml p24). On day 3 postinfection, the percentage of infected cells was evaluated by FACS analysis. A representative FACS dot plot (F), and results from three independent blood donors (G) are shown. Viral production was analyzed in cell culture supernatant by ELISA to detect the viral antigen p24 (H). *, *P* < 0.05; **, *P* < 0.005; ***, *P* < 0.0001. KD, knocked down.

Having shown that HIV-1 stimulates the expression and activity of HIF-1α, we decided to test the hypothesis that this transcription factor was involved in the promotion of HIV-1 replication. First, the expression of HIF-1α was silenced in Jurkat cells by lentiviral transduction with two short hairpin RNAs (shRNAs) specifically targeting this gene. Silencing was confirmed by quantitative PCR (qPCR) analysis of HIF-1α mRNA expression (Fig. 2A). Functional evidence of HIF-1α silencing was obtained by stimulating HIF-1α-silenced cells with the hypoxia mimetic CoCl_2_, a potent inductor of HIF-1α activity (18, 19). As expected, stimulation with CoCl_2_ triggered HIF-1α activity in control cells, but not in silenced cells (Fig. 2B). Finally, we verified that viability and proliferation rate of silenced cells were not altered (data not shown).

After infection of control and HIF-1α-silenced cells with HIV-1, we observed that viral replication was severely impaired in silenced cells, as revealed by analyzing the percentage of infected cells (Fig. 2C and D) and the amount of p24 released into the cell culture supernatant at day 3 postinfection (Fig. 2E). These results suggest that HIF-1α is required for HIV-1 replication. To confirm these observations in a more physiological model, the expression of HIF-1α was then silenced in primary CD4^+^ T cells. Following infection with HIV-1, both the percentage of infected cells (Fig. 2F and G) and the amount of p24 released into the cell culture supernatant (Fig. 2H) at day 3 postinfection were significantly reduced in HIF-1α-silenced CD4^+^ T cells. Finally, to further demonstrate the role of HIF-1α in HIV-1 replication, mock-infected or HIV-1-infected Jurkat cells were treated with CoCl_2_. Treatment with this hypoxia mimetic significantly increased the percentage of HIV-1-infected cells, suggesting that upregulation of HIF-1α activity promotes HIV-1 replication (Fig. S2A).

10.1128/mBio.00757-18.3 FIG S2  (A) Jurkat cells were infected with VSV-G-pseudotyped HIV-1-GFP (20 ng/ml p24) and subsequently stimulated with CoCl_2_ (100 µM). At day 3 p.i., the percentage of infected cells was determined by FACS analysis. Download FIG S2, TIF file, 0.1 MB. Copyright © 2018 Duette et al. 2018 Duette et al. This content is distributed under the terms of the Creative Commons Attribution 4.0 International license.

Altogether, the results presented here indicate that HIF-1α is required for HIV-1 replication and suggest that promotion of HIF-1α activity by HIV-1 stimulates viral replication.

### Promotion of HIF-1α activity by HIV-1 infection is triggered by viral nucleic acids.

**FIG 3:**
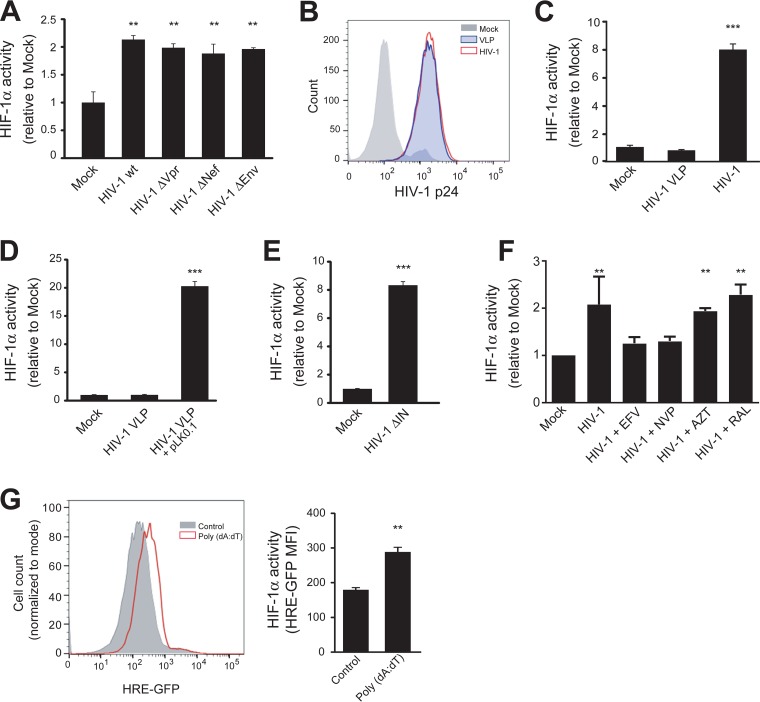
Promotion of HIF-1α activity by HIV-1 infection is triggered by viral nucleic acids. (A to F) HRE-GFP reporter Jurkat cells were infected or transduced with HIV-1wt or different viral mutants, and the expression levels of the HIF-1α reporter GFP were analyzed by FACS at 48 h p.i. (A) Cells were infected with HIV-1ΔVpr, HIV-1ΔNef, and HIV-1ΔEnv. HIV-1wt and mock infections were used as positive and negative controls, respectively. Representative data of four independent experiments are shown. (B and C) Cells were spinoculated (90 min) with either VSV-G-pseudotyped HIV-1 VLPs (empty) or VSV-G-pseudotyped HIV-1 and washed three times in PBS. (B) Viral particle entry was measured immediately after spinoculation by intracellular p24 staining. (C) Reporter GFP expression was determined by FACS analysis. Representative data of four independent experiments are shown. (D) Cells were spinoculated with VSV-G-pseudotyped HIV-1 VLPs (empty) or with HIV-1 VLPs containing the HIV-1-derived RNA from pLK0.1. Mock-infected cells were used as a control. Representative data of three independent experiments are shown. (E) Cells were spinoculated with a VSV-G-pseudotyped integrase-deficient HIV-1 mutant (HIV-1ΔIN). Mock spinoculation was used as a control. Representative data of five independent experiments are shown. (F) Cells were infected with HIV-1 in the presence of vehicle, EFV, NVP, AZT, and RAL. Pooled data from three independent experiments are shown. (G) HeLa HRE-GFP reporter cells were transfected with poly(dA-dT). Cells exposed to transfection reagent without DNA were used as a negative control. (Left) Histogram overlays show the expression of the reporter GFP from a representative experiment. (Right) Average plus SD of the MFI of GFP is shown (*n* = 4). **, *P* < 0.005; ***, *P* < 0.0001.

Our results showing that HIV-1 infection enhances the transcription and activity of HIF-1α in nonhypoxic conditions (Fig. 1) raises the hypothesis that a viral component, rather than hypoxia, is responsible for triggering HIF-1α activity in CD4^+^ T cells. To evaluate this hypothesis, we analyzed the role of a set of viral proteins that have been previously shown to modulate different cell signaling pathways. Jurkat HRE-GFP cells were infected with vesicular stomatitis virus G protein (VSV-G)-pseudotyped HIV-1 particles lacking the viral protein Vpr (previously shown to induce HIF-1α transcription in a human microglial cell line [20] and in a macrophage cell line [21]), Nef, or Env. The deficiency of any of the three viral proteins did not affect the ability of HIV-1 to induce HIF-1α activity (Fig. 3A), ruling out their involvement in the induction of HIF-1α activity in infected CD4^+^ T cells.

To determine whether viral entry, HIV-1 structural proteins, or viral nucleic acids were involved in triggering HIF-1α, we produced “empty” (without viral RNA) HIV-1 virus-like particles (VLPs). These VLPs were produced by expressing Gag/Pol in 293T cells, together with the plasmid coding for VSV-G protein, which was used to pseudotype VLPs to increase their transduction efficiency. Either Jurkat cells or Jurkat-HRE reporter cells were spinoculated with these nonreplicative particles to ensure high rates of uptake. For a positive control, we infected cells with wild-type HIV-1. The uptake of VLPs and HIV-1 particles was similar (Fig. 3B). However, “empty” VLPs did not activate the HIF-1α pathway (Fig. 3C), suggesting the following. (i) Viral entry alone is not enough to induce HIF-1α activity. (ii) Viral nucleic acids are required. To confirm the requirement of viral nucleic acids, we produced HIV-1 VLPs that, instead of containing the viral genome, carried the RNA derived from pLK0.1, a lentiviral vector for small interfering RNA (siRNA) expression (22). The RNA carried by these VLPs can be reverse transcribed in the recipient cell, generating cDNA that does not code for any viral protein. Whereas empty VLPs did not induce the activity of HIF-1α, VLPs containing the pLK0.1-derived RNA induced HIF-1α transcriptional activity (Fig. 3D). These results further suggest that the presence of either viral RNA or reverse-transcribed DNA is required for the induction of HIF-1α activity.

To evaluate whether viral DNA integration is required for eliciting the HIF-1α pathway, we spinoculated Jurkat HRE-GFP cells with a high multiplicity of infection (MOI) of integrase-defective HIV-1 (HIV-1ΔIN). This mutant virus is able to produce viral cDNA—albeit with lower efficiency (Fig. S3A)—but it is unable to proceed further with the viral replication cycle (23, 24). The strong induction of GFP expression by the IN-defective HIV-1 mutant (Fig. 2E) indicates that integration of the viral genome is not required to induce HIF-1α transcriptional activity and further supports the notion that viral nucleic acids are the viral component responsible for this effect.

10.1128/mBio.00757-18.4 FIG S3  (A) Jurkat cells were infected with HIV-1wt or HIV-1ΔIN or mock infected for 8 h. Production of viral dsDNA was quantified by PCR using two sets of specific primers that amplify two fragments of the HIV-1 long terminal repeat (LTR) (23). Primers were designed to detect intermediate (U3 to U5) and late (R-gag) products of reverse transcription. PCR products were separated on 1% agarose gel and visualized by ethidium bromide staining. (B) Efficacy of antiretroviral drugs used in the study to inhibit HIV-1 replication. Jurkat cells were infected with HIV-1 in the presence or absence of antiretroviral drugs (EFV, NVP, RAL, or AZT). Inhibition of HIV-1 replication was confirmed by intracellular p24 staining and FACS analysis on day 2 p.i. The percentage of infected cells is shown as a percentage of p24-positive cells. (C to E) CD4^+^ T cells isolated from blood samples from healthy donors were activated and subsequently infected with HIV-1ΔIN or mock infected. (C) Glucose uptake was evaluated by incubating cells for 30 min with the fluorescent glucose analog 2-NBDG (300 µM) in culture medium. Following washing, cells were analyzed by FACS. Representative data of three independent experiments are shown. (D) LDH activity was evaluated after cell lysis by measuring the reduction of tetrazolium salt to red formazan by an enzymatic reaction dependent on the amount of LDH present in the cell lysate. Red formazan absorbance was measured at 490 nm using a plate-reading spectrophotometer. Representative data of three independent experiments are shown. (E) The pH of the culture medium from infected and mock-infected cells was quantified as a proxy for glycolysis (acidification due to lactic acid production). (F to N) The cGAS/IFI16/STING pathway is not involved in triggering HIF-1α activity by HIV-1 infection. (F) The expression of cGAS was silenced in HRE-GFP reporter Jurkat cells. The efficiency of silencing was assessed by qPCR. (G) Control or cGAS-silenced HRE-GFP Jurkat reporter cells were infected with HIV-1 or mock infected. HIF-1α transcriptional activity was determined by FACS analysis of GFP expression. (H) The expression of IFI16 was silenced in HRE-GFP reporter Jurkat cells. The efficiency of silencing was assessed by qPCR. (I) Control or IFI-16-silenced HRE-GFP Jurkat reporter cells were infected with HIV-1 or mock infected. HIF-1α transcriptional activity was determined by FACS analysis of GFP expression. (J) The expression of STING was silenced in HRE-GFP reporter Jurkat cells. The efficiency of silencing was assessed by qPCR. (K) Control or STING-silenced HRE-GFP Jurkat reporter cells were infected with HIV-1 or mock infected. HIF-1α transcriptional activity was determined by FACS analysis of GFP expression. (L) The expression of STING was silenced in HRE-GFP reporter HeLa cells that were subsequently stimulated with poly(dA-dT) or with transfection reagent without nucleic acids. HIF-1α transcriptional activity was determined by FACS analysis of GFP expression. (M) HEK 293T HRE-GFP reporter cells were transfected with poly(dA-dT). Cells exposed to transfection reagent without DNA were used as a negative control. Histogram overlays show the expression of the reporter GFP from a representative experiment. (N) Pooled data from two independent experiments are shown. Representative data of three independent experiments are shown. *, *P* < 0.05; **, *P* < 0.005; ***, *P* < 0.0001; n.s., not significant. Download FIG S3, TIF file, 1.1 MB. Copyright © 2018 Duette et al. 2018 Duette et al. This content is distributed under the terms of the Creative Commons Attribution 4.0 International license.

To evaluate whether incoming viral RNA or reverse-transcribed cDNA were responsible for inducing the HIF-1α pathway, we infected the reporter cell line with wild-type HIV-1 (HIV-1wt) (NL4-3) in the presence of various antiretroviral agents. We used three reverse transcriptase inhibitors, efavirenz (EFV), nevirapine (NVP), and azidothymidine (AZT). Both EFV and NVP are allosteric inhibitors of the reverse transcriptase that blocks the activity of the enzyme, thus preventing the formation of cDNA (25). In contrast, AZT is a nucleoside reverse transcriptase inhibitor that allows the accumulation of short reverse transcripts (25–27). In addition, we used raltegravir (RAL), an integrase inhibitor. As expected, all drugs inhibited HIV-1 replication (Fig. S3B). Interestingly, treatment with RAL and AZT did not block the increase in the activity of HIF-1α induced by HIV-1 infection, while the treatment with EFV and NVP markedly inhibited the HIV-1-mediated induction of HIF-1α (Fig. 3F). These results suggest that reverse-transcribed DNA generated during HIV-1 replication is a key factor for triggering the HIF-1α signaling pathway.

Results presented above indicate that HIV-1 DNA integration (and therefore replication) is not required to trigger HIF-1α activity in CD4^+^ T cells. To evaluate whether infection with the IN-deficient mutant was also able to trigger glycolysis, we infected CD4^+^ T cells with this HIV-1 mutant and analyzed their glycolytic activity. We observed that infection with this viral mutant induced glucose uptake (Fig. S3C), LDH activity (Fig. S3D) and extracellular acidification (Fig. S3E).

Finally, to evaluate whether the induction of HIF-1α activity by cytosolic viral nucleic acids was restricted to HIV-1 or was a more extended phenomenon, we transfected HeLa HRE-GFP reporter cells with poly(dA-dT) ⋅ poly(dT-dA) [poly(dA-dT)] (a synthetic analog of double-stranded DNA [dsDNA]). We constructed the HeLa HRE-GFP cell line used because transfection of Jurkat cells is technically challenging. Transfection with the dsDNA analog induced HIF-1α activity (Fig. 3G), suggesting that cytosolic dsDNA is capable of triggering HIF-1α activity, irrespective of its sequence. Surprisingly, silencing of three key molecules in the cytosolic DNA sensing pathway (cyclic GMP-AMP synthase [cGAS], IFI16, and STING [*st*imulator of *in*terferon *g*enes]) did not inhibit the HIV-1-mediated induction of HIF-1α activity (Fig. S3F to K). Likewise, transfection of STING-silenced HeLa cells with poly(dA-dT) promoted HIF-1α activity (Fig. S3L). Finally, we transfected HRE-GFP reporter HEK 293T cells, which lack the endogenous STING pathway, with poly(dA-dT). Despite the fact that these cells have a defective cytosolic dsDNA sensing pathway, HIF-1α activity was increased in 293T cells (Fig. S3M and N). Altogether, these results suggest that reverse-transcribed DNA produced during the HIV-1 replication cycle triggers the HIF-1α pathway through a cGAS/IFI16/STING-independent pathway, resulting in an increase in the glycolytic activity of CD4^+^ T cells.

### Induction of HIF-1α activity by HIV-1 infection depends on the production of mitochondrial ROS triggered by cytosolic viral dsDNA.

**FIG 4:**
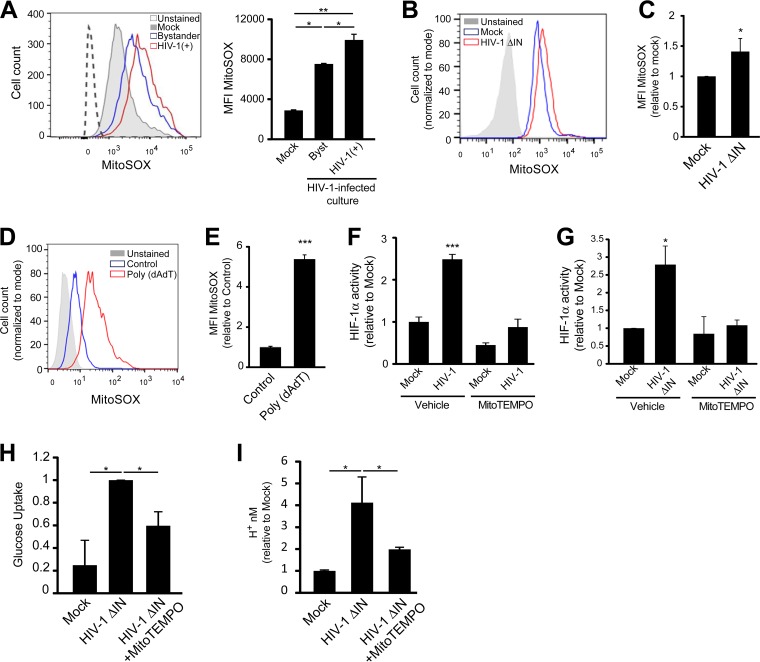
Induction of HIF-1α activity by HIV-1 infection depends on the production of mitochondrial ROS triggered by the cytosolic viral dsDNA. (A) CD4^+^ T cells isolated from blood samples from healthy donors were activated and subsequently infected with VSV-G-pseudotyped HIV-1-GFP or mock infected. mtROS production was measured using MitoSOX at day 2 postinfection in mock-infected cells (gray histogram), bystander cells (blue histogram) and HIV-1-infected (red histogram) CD4^+^ T cells. Unstained control is shown (filled gray histogram). Histograms from a representative experiment, and average MFI (*n* = 3) are shown. (B and C) mtROS production was measured in HIV-1ΔIN-infected and mock-infected Jurkat cells at 24 h postinfection. A representative histogram (B) and pooled data from three independent experiment are shown (C). (D and E) HeLa cells were transfected with poly(dA-dT), and mtROS production was measured at 24 h posttransfection. Cells exposed to transfection reagent without DNA were used as a negative control. A representative histogram (D) and pooled data from three independent experiments are shown (E). (F and G) To evaluate the contribution of mtROS on the promotion of HIF-1α activity, Jurkat HRE-GFP reporter cells were infected with HIV-1 (F) or HIV-1ΔIN (G), and after infection, the cells were incubated in the presence or absence of MitoTEMPO (500 µM). Two days postinfection, GFP expression was measured by FACS. Pooled data from three independent experiments are shown. (H and I) Activated primary CD4^+^ cells were infected with HIV-1ΔIN, and after infection, the cells were incubated in the presence or absence of MitoTEMPO (500 µM). Three days postinfection, glucose uptake analysis using the GlucCell assay (H) and pH quantification (I) were performed as a proxy for glycolysis. Pooled data from three independent experiment are shown. *, *P* < 0.05; **, *P* < 0.005; ***, *P* < 0.0001.

It has been previously shown that microbial pathogen-associated molecular patterns (PAMPs) trigger the production of mtROS by signaling through different Toll-like receptors (28). To gain further insight into the causes underlying the induction of HIF-1α during HIV-1 infection of CD4^+^ T cells, we evaluated the hypothesis that by inducing mtROS, HIV-1 triggers HIF-1α activity. We first evaluated mtROS production by using the probe MitoSOX, which specifically detects mitochondrial superoxide anion. We observed that both infected and bystander cells exhibited a considerable increase in mitochondrial superoxide anion content (Fig. 4A). Infection of Jurkat cells with the integrase-deficient HIV-1 mutant also induced an increase in mtROS production, indicating that viral integration and transcription are not required to induce this effect (Fig. 4B and C). To determine whether cytosolic dsDNA was responsible for triggering mtROS, we transfected the HeLa HRE-GFP reporter cell line with poly(dA-dT) and analyzed mtROS content. Interestingly, we observed that this short dsDNA fragment was capable of triggering mtROS production (Fig. 4D and E), suggesting that viral dsDNA generated during HIV-1 replication is the main stimulus for the induction of this oxidative compound in mitochondria.

To evaluate whether mtROS was responsible for triggering HIF-1α activity, we infected the Jurkat HRE-GRP reporter cell line, and MitoTEMPO, a specific quencher of mtROS, was then added to the culture. Addition of MitoTEMPO to cells infected with either HIV-1wt (Fig. 4F) or integrase-deficient HIV-1 (Fig. 4G) abolished the induction of HIF-1α activity triggered by infection but did not affect the percentage of infected cells (~46% under all conditions). As expected from the results presented above, inhibition of mtROS, and consequently, the induction of HIF-1α activity by the addition of MitoTEMPO, prevented the increase of glycolytic activity triggered by infection with the integrase-deficient HIV-1 (Fig. 4H and I).

### HIV-1 infection promotes the release of extracellular vesicles that induce HIF-1α activity in bystander cells.

**FIG 5:**
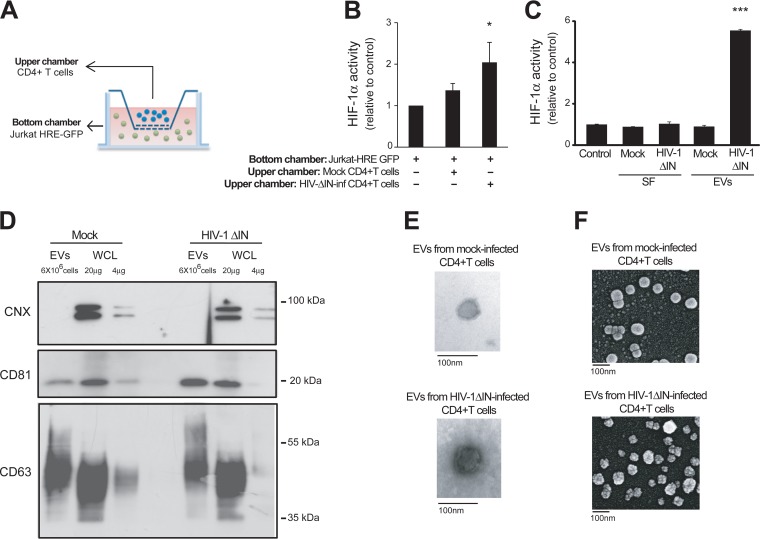
HIV-1 infection induces the extracellular vesicle-mediated propagation of HIF-1α activity to bystander cells. (A and B) In transwell experiments, CD4^+^ T cells isolated from blood samples from healthy donors were activated through stimulation with anti-CD3/CD28/CD2 antibody-coated beads for 48 h. A total of 6 × 10^6^ cells were either mock infected or infected with VSV-G-pseudotyped HIV-1ΔIN. On day 2 postinfection, cells (5 × 10^5^) were placed in the top chamber of a transwell (0.45 µm) and cocultivated with the Jurkat HRE-GFP cells (bottom chamber, 2 × 10^5^ cells). GFP expression of reporter cells was evaluated 2 days later. (A) Schematic representation of the experiment. (B) FACS analysis of pooled data from two independent experiments. (C) EVs and soluble fraction (SF) of cell culture supernatant of mock-infected or HIV-1ΔIN-infected primary CD4^+^ T cells were separated at day 2 p.i. by differential centrifugation. The EV pellet was resuspended in the same starting volume of medium. Jurkat HRE-GFP cells were incubated with either the SF or the isolated EVs for 48 h. FACS analysis of the MFI of GFP from a representative experiment is shown (*n* = 4). (D to F) Characterization of EVs released by mock-infected and HIV-1ΔIN-infected CD4^+^ T cells. (D) Presence of CD63, CD81 (canonical EV markers), and calnexin (CNX) (ER marker) was determined by immunoblotting of protein from isolated EVs and whole-cell lysates (WCL). EVs produced by equal numbers (6 × 10^6^) of mock-infected or HIV-1ΔIN-infected cells were analyzed together with WCL (20 and 4 µg). (E and F) Visualization of EVs by TEM (E) and SEM (F) was performed. *, *P* < 0.05; ***, *P* < 0.0001.

Results presented in Fig. 1 and 3 suggest that HIV-1 infection induces HIF-1α activity not only in productively infected cells but also in noninfected bystander cells. To further explore the mechanism underlying the induction of bystander effect, we cocultured HIV-1ΔIN-infected primary CD4^+^ T cells placed in the upper chamber of a transwell (0.4 µm) with HIF-1α reporter Jurkat cells present in the bottom chamber. The size of this pore precluded cell-cell contact but allowed the passage of soluble factors and extracellular vesicles (EVs) (Fig. 5A). Infections were done with HIV-1ΔIN because this viral mutant is capable of inducing HIF-1α activity in infected cells (Fig. 3E) but does not produce viral progeny (24). The induction of HIF-1α activity in target reporter cells, even when separated by a transwell (Fig. 5B), indicated that this induction was mediated by a secreted factor produced by HIV-1ΔIN-infected CD4^+^ T cells. We then investigated whether this effect was mediated by a soluble factor or EVs. Separation of HIV-1 and EVs is extremely difficult, because they share most physicochemical properties (29). Thus, utilization of the integrase-deficient mutant makes this virus particularly suitable for studying EVs produced by HIV-1-infected cells. We infected CD4^+^ T cells with HIV-1ΔIN and 48 h later, cell culture supernatants were differentially centrifuged to obtain EV-free soluble fractions and pelleted EVs, removing cells and large debris. Conditioned medium or purified EVs from mock-infected CD4^+^ T cells were used as controls. The EV-depleted supernatant (soluble fraction [SF]) from either HIV-1ΔIN or mock-infected CD4^+^ T cells did not induce HIF-1α activity, ruling out the contribution of soluble factors (Fig. 5C). In contrast, pelleted EVs produced by HIV-1ΔIN-infected cultures strongly induced HIF-1α activity in reporter cells (Fig. 5C). We confirmed the lack of infectious virus in our EV preparations by incubating EVs with the HIV-1 reporter cell line GHOST (Fig. S4A).

10.1128/mBio.00757-18.5 FIG S4  (A) The lack of virus production by HIV-1ΔIN-infected CD4^+^ T cells was confirmed using GHOST cells. GHOST cells were incubated with EVs produced by CD4^+^ T cells infected with HIV-1ΔIN. Infection of GHOST cells with HIV-1wt or HIV-1ΔIN or mock-infected cells were used as a control. The percentage of GFP-positive cells was assessed at 24 h p.i. by FACS analysis. Download FIG S4, TIF file, 0.1 MB. Copyright © 2018 Duette et al. 2018 Duette et al. This content is distributed under the terms of the Creative Commons Attribution 4.0 International license.

The EV preparations were then characterized by detection of the canonical EV markers CD63 and CD81 by immunoblotting (Fig. 5D). The EV-excluded endoplasmic reticulum (ER) marker calnexin was, as expected, present in cell lysates, but not in EVs (Fig. 5D). EV visualization by transmission electron microscopy (TEM) (Fig. 5E) and scanning electron microscopy (SEM) (Fig. 5F) revealed that EVs secreted by both HIV-1ΔIN-infected and mock-infected CD4^+^ T cells had a spherical shape and a diameter that ranged from 50 to 200 nm. These results suggest that infection with HIV-1ΔIN induces the secretion of functionally different EVs compared with EVs produced by mock-infected cells.

### HIV-1-induced EVs promote the production of cytokines by CD4^+^ T cells and macrophages through a HIF-1α-dependent pathway.

**FIG 6:**
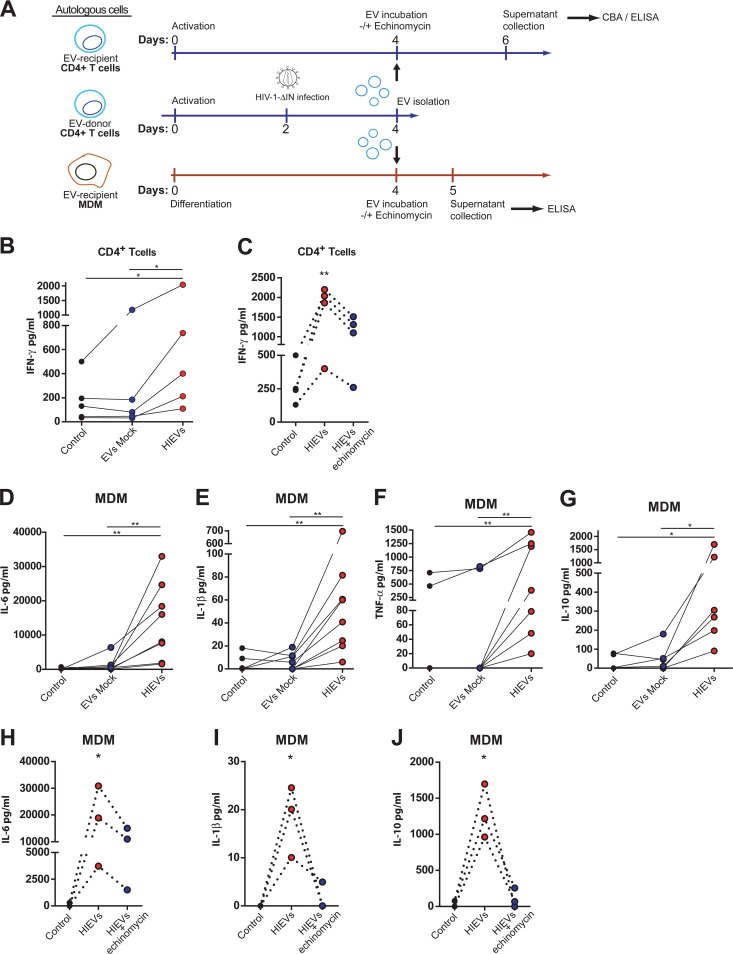
EVs released by HIV-1-infected cells promote HIF-1α-mediated secretion of proinflammatory cytokines in CD4^+^ T cells and macrophages. (A) Schematic representation of the experimental design. HIEVs or EVs from 6 × 10^6^ mock-infected CD4^+^ T cells were purified on day 2 p.i. and added to autologous uninfected activated CD4^+^ T cells or macrophages. Cytokine secretion was quantified by ELISA or cytokine bead array (CBA). (B) HIEVs or EVs from mock-infected cells were added to autologous CD4^+^ T cells. Secretion of IFN-γ by CD4^+^ T cells that had received EVs was assessed by ELISA. (C) IFN-γ production by CD4^+^ T cells exposed to HIEVs in the presence or absence of echinomycin (1 nM) was determined by ELISA. (D to G) HIEVs and EVs from mock-infected CD4^+^ T cells were added to autologous macrophages, and production of IL-6 (D), IL-1β (E), TNF-α (F), and IL-10 (G) was measured by ELISA. Results from individual experiments using independent blood donors are shown. (H to J) Production of IL-6 (H), IL-1β (I), and IL-10 (J) by monocyte-derived macrophages (MDM) exposed to HIEVs and treated with echinomycin (1 nM) was measured by ELISA. *, *P* < 0.05; **, *P* < 0.005.

Considering that HIF-1α plays a critical role in the development of inflammatory immune responses (10), we decided to silence HIF-1α expression in primary CD4^+^ T cells. The silenced cells were infected with HIV-1, and production of the proinflammatory cytokine gamma interferon (IFN-γ) was analyzed by an enzyme-linked immunosorbent assay (ELISA). We observed that HIF-1α-silenced cells produced significantly less interferon than control cells, showing that this transcription factor plays a direct role in CD4^+^ T cell-mediated inflammation (Fig. S5A). Next, we evaluated whether the HIV-1-induced EV (HIEV)-mediated induction of HIF-1α in bystander CD4^+^ T cells stimulated the secretion of cytokines. Uninfected CD4^+^ T cells were activated, and 48 h later, HIEVs or EVs produced by control cells were added to the culture medium (Fig. 6A). Production of interleukin 17A (IL-17A), IFN-γ, IL-2, IL-4, tumor necrosis factor alpha (TNF-α), IL-6, and IL-10 was evaluated 2 days later. Remarkably, we observed that CD4^+^ T cells incubated with HIEVs secreted considerably larger amounts of IFN-γ than cells incubated with EVs produced by mock-infected CD4^+^ T lymphocytes (Fig. 6B). Moreover, we also observed that HIEVs stimulated IL-17A production in cells from some donors, but not by others (not shown). Interestingly, if CD4^+^ T cells that had received EVs were pretreated with echinomycin to inhibit HIF-1α activity, the HIEV-mediated induction of IFN-γ secretion was significantly reduced, but not abolished (Fig. 6C). This observation suggests that HIEVs increase the amount of secreted IFN-γ by inducing HIF-1α activity in bystander cells.

10.1128/mBio.00757-18.6 FIG S5  Silencing HIF-1α expression with shRNA in CD4^+^ T cells impairs the HIV-1 mediated secretion of EVs both directly (A) and indirectly (B). Download FIG S5, TIF file, 0.3 MB. Copyright © 2018 Duette et al. 2018 Duette et al. This content is distributed under the terms of the Creative Commons Attribution 4.0 International license.

In addition to T cell dysfunction, chronic inflammation mediated by macrophages is another hallmark of HIV-1 infection that is associated with development of non-AIDS-related diseases. Thus, we aimed at assessing whether HIEVs promoted cytokine production in macrophages in a HIF-1α-dependent manner. Monocyte-derived macrophages (MDM) were prepared, and on day 4, HIEVs or control EVs produced by autologous CD4^+^ T cells were added to the culture medium. Remarkably, we observed that HIEVs, but not control EVs, promoted the secretion of the cytokines IL-6 (Fig. 6D), IL-1β (Fig. 6E), and TNF-α (Fig. 6F), but not IL-12 (not shown). Secretion of these inflammatory cytokines was accompanied by the secretion of IL-10 (Fig. 6G). Pharmacological inhibition of HIF-1α activity in macrophages significantly reduced the secretion of IL-6 (Fig. 6H) and abolished secretion of IL-1β (Fig. 6I) and IL-10 (Fig. 6J). The secretion of TNF-α by cells in which HIF-1α activity was pharmacologically inhibited yielded no consistent results (not shown). These results indicate that HIEVs released by CD4^+^ T cells have the ability to promote the secretion of different cytokines in both HIF-1α-dependent and -independent manners by activated CD4^+^ T cells and by macrophages.

### Induction of HIF-1α activity triggered by HIV-1-derived nucleic acids is required for the production of inflammatory HIEVs.

**FIG 7:**
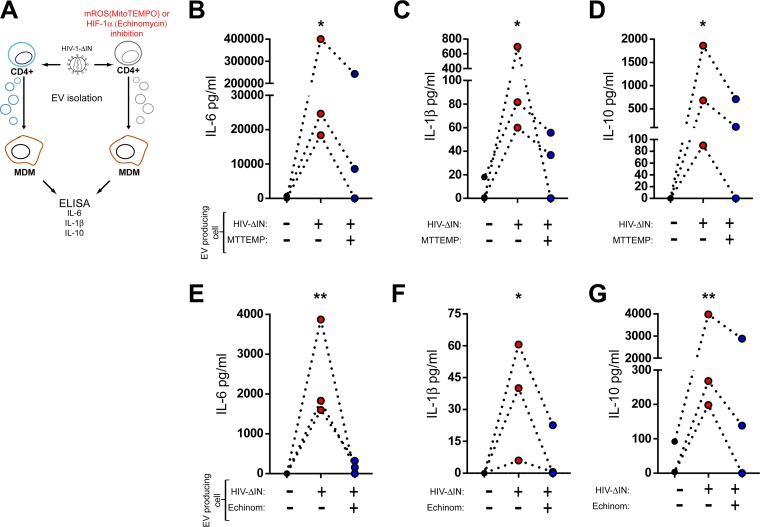
Induction of HIF-1α activity triggered by HIV-1-derived nucleic acids is a prerequisite for the production of inflammatory HIEVs. (A) Schematic representation of the experimental design for panels B to G. (B to D) Primary CD4^+^ T cells were activated, infected (+) or not infected with HIV-1ΔIN, and subsequently treated with MitoTEMPO (MTTEMP) (500 µM) (+). Vesicles were purified, extensively washed, and added to autologous macrophage cultures. The production of IL-6 (B), IL-1β (C), and IL-10 (D) was evaluated 24 h later. (E to G) Primary CD4^+^ T cells were activated, infected or not infected with HIV-1 ΔIN and subsequently treated with echinomycin (Echinom) (1 nM). Vesicles were purified, extensively washed, and added to autologous macrophage cultures. The production of IL-6 (E), IL-1β (F), and IL-10 (G) was evaluated 24 h later. *, *P* < 0.05; **, *P* < 0.005.

We next asked whether HIF-1α was necessary for the production of HIEVs with proinflammatory activity. Taking into consideration that HIF-1α activity is induced in a mitochondrial ROS-dependent manner, we first treated HIV-1ΔIN-infected CD4^+^ T cells with MitoTEMPO to reduce ROS generation. EVs purified from the supernatant of MitoTEMPO-treated CD4^+^ T cells were then added to macrophage cultures, and cytokine production was analyzed 48 h later (Fig. 7A). Production of IL-6 (Fig. 7B), IL-1β (Fig. 7C), and IL-10 (Fig. 7D) was significantly inhibited in cells treated with MitoTEMPO, suggesting that mitochondrial ROS production in CD4^+^ T cells is required for production of EVs. To further confirm the involvement of HIF-1α in the generation of proinflammatory EVs, infected CD4^+^ T cells were treated with echinomycin (Fig. 7E to G). Two days after infection and addition of the pharmacological inhibitor, HIEVs were purified and added to macrophage cultures. HIEVs from CD4^+^ T cells exposed to vehicle triggered the secretion of IL-6, IL-1β, and IL-10. This response was significantly decreased if HIF-1α activity in EV-producing cells was inhibited by treatment with echinomycin (Fig. 7E to G). Finally, analysis of the induction of IL-6 by HIEVs produced by HIF-1α-silenced cells confirmed that the activity of this transcription factor in CD4^+^ T cells is required for the production of proinflammatory EVs (Fig. S5B).

### Extracellular vesicles purified from plasma samples from HIV-1-infected individuals trigger macrophage-mediated inflammation and HIF-1α activity.

**FIG 8:**
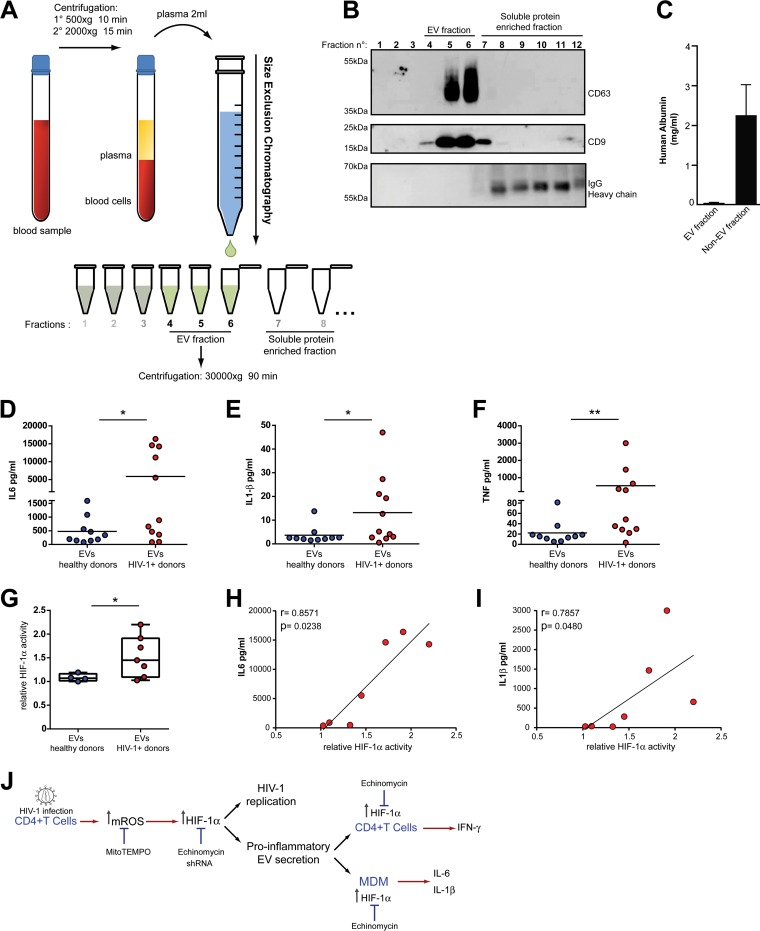
EVs isolated from plasma samples from HIV-1-infected individuals are proinflammatory and induce HIF-1α activity. (A) Schematic diagram depicting the procedure to isolate EVs from human plasma by size exclusion chromatography (SEC). (B) Immunoblot analysis of the first 12 fractions obtained by SEC. The presence of the EV markers CD63 and CD9 and the presence of IgG was analyzed. (C) Quantification of human albumin in the pooled EV fraction (fractions 4 to 6) and in the pooled non-EV fraction (fractions 7 to 12). (D to F) EVs isolated from plasma samples from either healthy controls or HIV-1 individuals were added to monocyte-derived macrophages, and production of the proinflammatory cytokines IL-6, IL-1β, and TNF-α was analyzed by ELISA on day 2 after addition of the EVs. (G) The ability of EVs isolated from plasma to stimulate HIF-1α activity was analyzed using the HeLa HRE-GFP reporter cell line. EVs isolated from plasma samples from a subset of HIV-1-infected individuals and healthy donors and the reporter cell line were incubated for 48 h, and the HIF-1α-driven expression of GFP was analyzed by FACS. (H and I) Correlation between the HIF-1α-inducing activity of EVs and the amounts of IL-6 (H) and IL-1β (I) secreted by stimulated macrophages. (J) Working model proposing that mitochondrial ROS (mtROS) production triggered by HIV-1 infection induces HIF-1α activity, resulting in an increase in viral replication and the production of HIEVs which, in turn, induce HIF-1α activity and inflammation in bystander cells. *, *P* < 0.05; **, *P* < 0.005.

In order to gain further insight into the physiological relevance of our findings, we decided to isolate and analyze the function of EVs present in the plasma of HIV-1-infected individuals. EVs from 2-ml plasma samples from HIV-1-positive (Table 2) and HIV-1-negative individuals were isolated by size exclusion chromatography, followed by centrifugation of EVs and resuspension in phosphate-buffered saline (PBS) (Fig. 8A). The presence of EVs in each fraction was determined by immunoblotting to detect EV markers CD63 and CD9. The efficiency of the purification process was determined by analyzing the presence of soluble components of the plasma. We observed that fractions 4, 5, and 6 showed an enrichment of the EV markers CD63 and CD9 (Fig. 8B). Conversely, these fractions were negative for the presence of soluble proteins IgG (Fig. 8B), albumin (Fig. 8C), and fibrinogen (Fig. S6). Thus, we pooled these three fractions from each sample for subsequent functional studies. Since viral particles will very likely copurify with EVs, we decided to use plasma samples from cART-treated individuals with undetected viral loads.

10.1128/mBio.00757-18.7 FIG S6  Immunoblot analysis of fractions 3 to 11 obtained by size exclusion chromatography (SEC) of 2-ml human plasma samples. The presence of the EV marker CD9 and the presence of fibrinogen-γ chain were analyzed. Download FIG S6, TIF file, 0.4 MB. Copyright © 2018 Duette et al. 2018 Duette et al. This content is distributed under the terms of the Creative Commons Attribution 4.0 International license.

**TABLE 2:** Clinical characteristics of study group of HIV-positive patients on cART

Patient	Age (yr)	% CD4^+ ^T cells	CD4^+^ T cell count	Sex^a^
00060	41	28	814	F
00061	35	43	1,082	M
00062		16	495	
00063		40	540	
00072		33	524	
00073	28	44	863	F
00074	41	37	838	F
00078	24	34	712	M
00090	30	27	1,028	M
00096	36	ND^b^	ND	F
00097	21	26	674	M

a F, female; M, male.

b ND, not determined.

To analyze the proinflammatory activity of plasma-derived EVs, isolated vesicles were incubated with MDM for 24 h, and the production of proinflammatory cytokines in cell culture supernatants was evaluated by cytokine bead array (CBA) (Fig. 8D to F). We observed that EVs from HIV-1-infected individuals induced significantly higher levels of IL-6 (Fig. 8D), IL-1β (Fig. 8E), and TNF-α (Fig. 8F) compared to EVs from healthy donors. These results indicate that during HIV-1 infection, EVs present in plasma are capable of triggering proinflammatory cytokine release in macrophages.

Taking into consideration that the proinflammatory activity of EVs produced by CD4^+^ T cells infected *in vitro* with HIV-1 depends on the induction of HIF-1α activity in macrophages (Fig. 6), we then analyzed the ability of EVs present in the plasma of HIV-1-infected individuals to trigger HIF-1α activity in the reporter cell line, HeLa HRE-GFP. We observed that, like EVs produced *in vitro* by CD4^+^ T cells infected with HIV-1, EVs isolated from plasma samples from HIV-1-infected individuals triggered HIF-1α activity (Fig. 8G). Moreover, we observed that there was a positive correlation between the level of induction of HIF-1α activity and the secretion of IL-6 and IL-1β (Fig. 8H and I), suggesting that induction of HIF-1α activity by plasma EVs is associated with the production of these inflammatory cytokines.

Altogether, our results demonstrate that by inducing the production of mitochondrial ROS, HIV-1 infection enhances HIF-1α activity. Heightened activity of this transcription factor promotes viral replication and the release of proinflammatory HIEVs, which in turn stimulate secretion of proinflammatory cytokines by bystander macrophages and CD4^+^ T cells (Fig. 8J).

## DISCUSSION

In this study, we present three major results. First, we show that viral nucleic acids, in particular dsDNA generated during HIV-1 replication, induce the mitochondrial ROS-dependent stimulation of HIF-1α activity in CD4^+^ T cells, promoting HIV-1 replication. Second, we demonstrate that HIV-1 infection induces the HIF-1α-dependent secretion of proinflammatory HIV-1-induced EVs (HIEVs). These EVs, in turn, are capable of triggering the HIF-1α-dependent production of IFN-γ by bystander CD4^+^ T cells and IL-6/IL-1β by bystander macrophages. Third, we show that EVs isolated from plasma samples from HIV-1-infected individuals on combination antiretroviral therapy (cART) and with undetected viral loads can trigger HIF-1α activity and inflammation *ex vivo*. Overall, our results posit HIF-1α and HIEVs as key players in the pathogenesis of the inflammation that is associated with HIV-1 infection.

Regulation of HIF-1α activity in CD4^+^ T cells is a complex process. Hypoxia, T cell receptor (TCR) stimulation, and certain cytokines can trigger the activity of this transcription factor (30). Moreover, it has been suggested that mitochondrial ROS (mtROS) generated during T cell activation could also promote HIF-1α stabilization and activity. Herein, we show that by inducing the production of mitochondrial ROS, cytosolic dsDNA generated during HIV-1 replication promotes HIF-1α activity in CD4^+^ T cells. Accessory proteins expressed by HIV-1 do not participate in triggering the HIF-1α pathway, as revealed by experiments with viral mutants deficient for individual viral structural and accessory proteins. These results thus demonstrate that a viral pathogen-associated molecular pattern (PAMP) is responsible for triggering the HIF-1α pathway in CD4^+^ T cells and contrast with previous reports showing that the viral accessory protein Vpr is responsible for triggering HIF-1α induction in human microglial cells (20). This contrasting observation suggests that the mechanisms responsible for triggering HIF-1α during HIV-1 infection may be cell type specific.

Increased production of ROS in productively infected CD4^+^ T cells and in HIV-1-infected individuals have been previously reported (31). Indeed, increased ROS production during HIV-1 infection has been associated with CD4^+^ T cell apoptosis (32). Herein, we demonstrate that an additional outcome of ROS induction during HIV-1 infection is the stabilization of HIF-1α. As expected, the increase in HIF-1α activity is associated with the stimulation of glycolysis. Moreover, we show that HIF-1α is required for HIV-1 replication in primary CD4^+^ T cells. Future studies will explore the mechanisms underlying the enhancement of viral replication in CD4^+^ T cells by HIF-1α. Remarkably, we also show that heightened HIF-1α activity promotes the secretion of proinflammatory HIEVs from CD4^+^ T cells. These virus-induced EVs, but not EVs secreted by uninfected CD4^+^ T cells, are capable of inducing the activation of HIF-1α in bystander cells, thus propagating this cellular response from productively infected cells to other noninfected cells of the immune system. Along these lines, we present data showing that CD4^+^ T cells from HIV-1-infected individuals express higher levels of HIF-1α compared with the same cells from healthy individuals. This observation provides further evidence for the bystander induction of HIF-1α. Moreover, we show that EVs isolated from plasma samples from HIV-1-infected individuals trigger HIF-1α in recipient cells and, more importantly, induce the secretion of cytokines by macrophages. Although we do not yet know the exact cellular source(s) of plasma EVs that carry the proinflammatory activity in HIV-1-infected individuals, CD4^+^ T cells may well contribute.

EVs can mediate different types of intercellular communication processes by delivering nucleic acids, lipids and proteins into target cells (33). It has been recently reported that EVs secreted by HIV-1-infected cells contain trans-activation response element (TAR) RNA (34) and that this viral RNA can induce the production of proinflammatory cytokines by monocyte-derived macrophages (34, 35). Moreover, it has also been shown that EVs can mediate the transfer of dsDNA between cells (36). Although we did not analyze the nucleic acid content of HIEVs, the possibility that they contain TAR RNA or viral DNA and that these nucleic acids mediate the effects triggered by HIEVs is interesting and merits further investigation.

Previous studies performed with cancer cells have shown that, under hypoxic conditions, HIF-1α can stimulate the release of EVs (37) with protumoral activities. For instance, HIF-1α can induce the Rab22-dependent secretion of EVs by human breast cancer cells. These EVs, in turn, promote focal adhesion formation, invasion, and metastasis (16). Likewise, hypoxic multiple myeloma cells secrete elevated levels of miR-135b-containing exosomes, which enhance endothelial tube formation, thus promoting angiogenesis (38). Collectively, this evidence indicates that cancer-associated hypoxia modulates the amount and function of exosomes and other EVs. In agreement with these observations, our results show that the mtROS-dependent induction of HIF-1α activity by HIV-1-derived dsDNA confers a proinflammatory signature to EVs. Pharmacological inhibition of either mtROS or HIF-1α in CD4^+^ T cells abolished the production of EVs capable of promoting the secretion of proinflammatory cytokines by macrophages.

The EV-mediated induction of HIF-1α in bystander CD4^+^ T cells and macrophages is required for the secretion of proinflammatory cytokines by these cells. Indeed, pharmacological inhibition of HIF-1α activity in cells that had received EVs abrogated the HIEV-induced secretion of IFN-γ by CD4^+^ T cells and of IL-1β and IL-6 by macrophages. We therefore postulate that the EV-mediated induction of HIF-1α activity in bystander macrophages during the course of HIV-1 infection might play a role in the induction of non-AIDS-related complications of HIV-1-positive (HIV-1+) individuals that are strongly related to a sustained chronic inflammatory response. These results are consistent with previous reports showing that HIF-1α is required for inflammatory responses in macrophages (39).

We provide a body of evidence that indicate that dsDNA is responsible for triggering the mtROS/HIF-1α/EV response to HIV-1 infection. We show that HIF-1α activity is triggered by the following: (i) HIV-1-derived VLPs carrying an RNA that can be reverse transcribed but not by VLPs devoid of nucleic acids; (ii) an integrase mutant HIV-1, which can reverse transcribe its RNA but that fails to produce progeny virus; (iii) transfection with synthetic dsDNA [poly(dA-dT)]. Moreover, we show that the reverse transcription (RT) inhibitors efavirenz and nevirapine abolish the response to infection, further supporting the idea that reverse-transcribed DNA is the main trigger of HIF-1α activity. However, the exact pathway responsible for sensing this DNA has not yet been elucidated. We silenced the two cytosolic dsDNA sensors previously described to be active in CD4^+^ T cells in response to HIV-1 infection: cGAS and IFI16 (40, 41). Moreover, we used cells deficient in the signaling molecule STING. Yet in each of these cases, the response to HIV-1 infection and even to synthetic dsDNA were not altered. These observations open the possibility that another cytosolic dsDNA sensor could be responsible for detecting the DNA. Alternatively, another sensing pathway, such as the TLR-mediated pathway, could be responsible for detecting the dsDNA generated during HIV-1 infection. Finally, despite the fact that treatment with efavirenz and nevirapine severely impaired the HIV-1-mediated induction of HIF-1α, we cannot completely rule out the possibility that viral RNA is contributing to the promotion of HIF-1α activity.

HIV-1-positive individuals have elevated markers of systemic inflammation, which are strongly predictive of the risk of morbidity and mortality. It is well accepted that HIV-1-infected patients present increased levels of circulating lipopolysaccharide (LPS) and that this bacterial PAMP is responsible for activating monocytes and macrophages, thus contributing to chronic inflammation. Our results demonstrate critical and previously unrecognized roles of HIV-1-derived dsDNA, mtROS and HIF-1α in the development of inflammatory EVs during the course of HIV-1 infection. Remarkably, EVs are present in the circulation of HIV-1-infected individuals undergoing effective cART. These EVs could be responsible for the sustained HIF-1α levels observed in circulating CD4^+^ T cells.

Overall, our study shows that HIF-1α and EVs coordinately participate in HIV-1 pathogenesis, both by promoting viral replication and by inducing the secretion of extracellular vesicles. These HIF-1α-dependent EVs, in turn induce the secretion of cytokines following interaction with target macrophages. Modulation of either mtROS production or HIF-1α activity in cART-treated HIV-1-infected individuals could represent a therapeutic strategy to counterbalance inflammation during HIV-1 infection, thus reducing the risk of developing serious non-AIDS events.

## MATERIALS AND METHODS

See Text S1 in the supplemental material for additional Materials and Methods.

10.1128/mBio.00757-18.1 TEXT S1  Supplemental Materials and Methods. Download TEXT S1, DOCX file, 0.02 MB. Copyright © 2018 Duette et al. 2018 Duette et al. This content is distributed under the terms of the Creative Commons Attribution 4.0 International license.

### Cell lines, plasmids, and HIV-1 strains.

The human CD4^+^ T cell line Jurkat clone E6.1, HeLa cells, and GHOST cells were obtained from the NIH AIDS Reagent Program, Division of AIDS, NIAID, NIH.

HEK 293T cells were obtained from ATCC (CRL-11268).

NL4-3-IRES-eGFP (IRES stands for internal ribosomal entry site, and eGFP stands for enhanced green fluorescent protein), encoding full-length HIV-1 in the pBR322 backbone under the control of the viral long terminal repeat promoter, pBR-NL4-3 ΔVpu and pBR-NL4-3 ΔVpr, was kindly provided by F. Kirchhoff (Institute of Molecular Virology, Ulm University Medical Center, Ulm, Germany) (42). pBR-NL4-3 ΔNEF and pBR-NL4-3 ΔENV were kindly provided by O. Schwartz (Institut Pasteur, Paris, France). pNLX.Luc.R-ΔIN (Luc stands for luciferase) was kindly provided by A. Engelman (Dana-Farber Cancer Institute, USA).

HIV-1 primary isolates pCH077 (CXCR4-tropic), pREJO.c, and pTRJO.c (CCR5-tropic) and HIV-1 clones MN (CXCR4-tropic), BaL (CCR5-tropic), and RF (dual-tropic) were obtained from the NIH AIDS Reagent Program, Division of AIDS, NIAID, NIH.

The plasmid 5HRE-hCMV-d2EGFP (hCMV stands for human cytomegalovirus) (HRE-GFP) was kindly provided by Martin Brown and Thomas Foster (Addgene plasmid 46926).

Lentiviruses expressing short hairpin RNA (shRNA) were obtained from Sigma (Mission shRNA).

### Antibodies.

The following antibodies were used: fluorescein isothiocyanate (FITC)-labeled and phycoerythrin (PE)-labeled mouse anti-p24 (KC57-FITC or KC57-PE; Beckman Coulter), Alexa Fluor 594-labeled anti-mouse (Jackson ImmunoResearch), allophycocyanin (APC)-labeled anti-human Glut-1 antibody (MAb1418 clone [R&D Systems, USA]), mouse anti-HIF-1α-PE (clone 241812; R&D Systems, USA), mouse anti-human HIF-1α (monoclonal antibody [MAb] 54 clone, BD Transduction Laboratories), mouse anti-human CD81 (clone J5-81; BD Bioscience), mouse anti-CD63 (clone H5C6; BD Bioscience), rabbit anti-calnexin (Abcam), and mouse anti-human CD9 (clone M-L13; BD Bioscience).

### HIF-1α reporter cell lines.

To evaluate HIF-1α transcriptional activity, we generated Jurkat, HeLa, and HEK 293T stable reporter cell lines by electroporation or transfection of the 5HRE-hCMV-d2EGFP vector (HRE-GFP). Jurkat cells (5 × 10^6^ cells) were electroporated with 20 µg DNA at 0.26 V/960 µF in a Gene Pulser II electroporation system (Bio-Rad). After electroporation, cells were resuspended in RPMI 1640 medium supplemented with 10% fetal bovine serum (FBS) (Gibco), and 3 days later, G418 was added for selection of cells that incorporated the plasmid (Jurkat HRE-GFP).

HeLa and HEK 293T cells were transfected with the HRE-GFP vector. The cells were cultured in Dulbecco modified Eagle medium (DMEM) supplemented with 10% fetal calf serum (FCS). Three days later, G418 was added to HeLa cell cultures for selection of cells that incorporated the plasmid. To select cells expressing detectable amounts of the reporter GFP, cells were stimulated with CoCl_2_ (100 µM) for 24 h, and GFP-positive cells were sorted by fluorescence-activated cell sorting (FACS) sorted on a BD FACSAria Fusion sorter to obtain Jurkat, HeLa, and 293T HRE-GFP cells.

### Extracellular vesicle purification.

Cells were cultured in “exosome-depleted medium” (complete medium depleted of FBS-derived exosomes by overnight centrifugation at 100,000 × *g*) for 48 h. Live and dead cells as well as large debris were removed by successive centrifugations at 300 × *g* for 10 min and 2,000 × *g* for 10 min. After each centrifugation, the supernatant was transferred into a new tube, while the generated pellets were discarded. Extracellular vesicles (EVs) were pelleted by centrifugation at 30,000 × *g* for 1.5 h.

### Nucleic acid stimulation.

Cytosolic delivery of the repetitive synthetic double-stranded DNA sequence of poly(dA-dT) ⋅ poly(dT-dA) [poly(dA-dT)] (InvivoGen) (1 mg/ml) was achieved by transfection of HeLa HRE-GFP (7 × 10^4^ cells/well in 24-well plates) and HEK 293T-HRE cells using the X-TremeGene HP reagent (Roche).

### Isolation of EVs from human plasma.

Isolation of EVs from plasma was performed following the protocol published by Böing et al. (43) with some modifications. Briefly, whole blood drawn by venipuncture was collected in EDTA-containing vacutainer tubes (BD). Platelet-rich plasma (PRP) was obtained by centrifugation (300 × *g*, 10 min). PRP was supplemented with 200 nM prostaglandin I_2 _(PGI_2_) to avoid platelet activation and centrifuged (600 × *g*; 10 min) to obtain cell-free plasma. Plasma samples (2 ml) were loaded onto a homemade size exclusion chromatography column (resin, CL-2B from GE Healthcare; support, 12-ml empty cartridges with 20-mm hydrophobic frits from Applied Separations). Twelve fractions (1 ml each) were eluted using 0.9% NaCl–0.38% sodium citrate. Each fraction was analyzed for the presence of the EV markers CD63 and CD9 and the soluble components IgG and fibrinogen by immunoblotting. Additionally, the concentration of albumin was analyzed by turbidimetry using a commercial kit (Wiener, Albúmina AA). Finally, EV-containing fractions were pooled, concentrated by ultracentrifugation, resuspended in phosphate-buffered saline (PBS), and used within the same day for functional studies.

### Study participants.

HIV-1-positive (HIV-1+) participating individuals were recruited from the community and the Infectious Diseases Unit at The Alfred Hospital in Melbourne, Australia. Blood samples from individuals recruited in Melbourne were collected in citrate anticoagulant tubes and processed within 1 h of venipuncture to isolate and cryopreserve peripheral blood mononuclear cells (PBMCs).

To isolate plasma EVs, HIV+ individuals were recruited at the Fundación Huesped Medical Center in Buenos Aires, Argentina. Blood samples were collected in citrate anticoagulant tubes and processed within 1 h of venipuncture to isolate plasma for EV purification.

Healthy donors were voluntary blood donors at the Sanatorio Dr. Julio Mendez blood bank (Buenos Aires, Argentina). All healthy donors were individuals older than 18 years who had completed and passed a survey on blood donation and were screened for serological markers before being accepted as donors.
