# Supplementary figures and images for: Induction of HIF-1α by HIV-1 Infection in CD4+ T Cells Promotes Viral Replication and Drives Extracellular Vesicle-Mediated Inflammation
Source: mBio. 2018 Sep 11;9(5):e00757-18. doi: 10.1128/mBio.00757-18 (PMC6134101; doi:10.1128/mBio.00757-18)

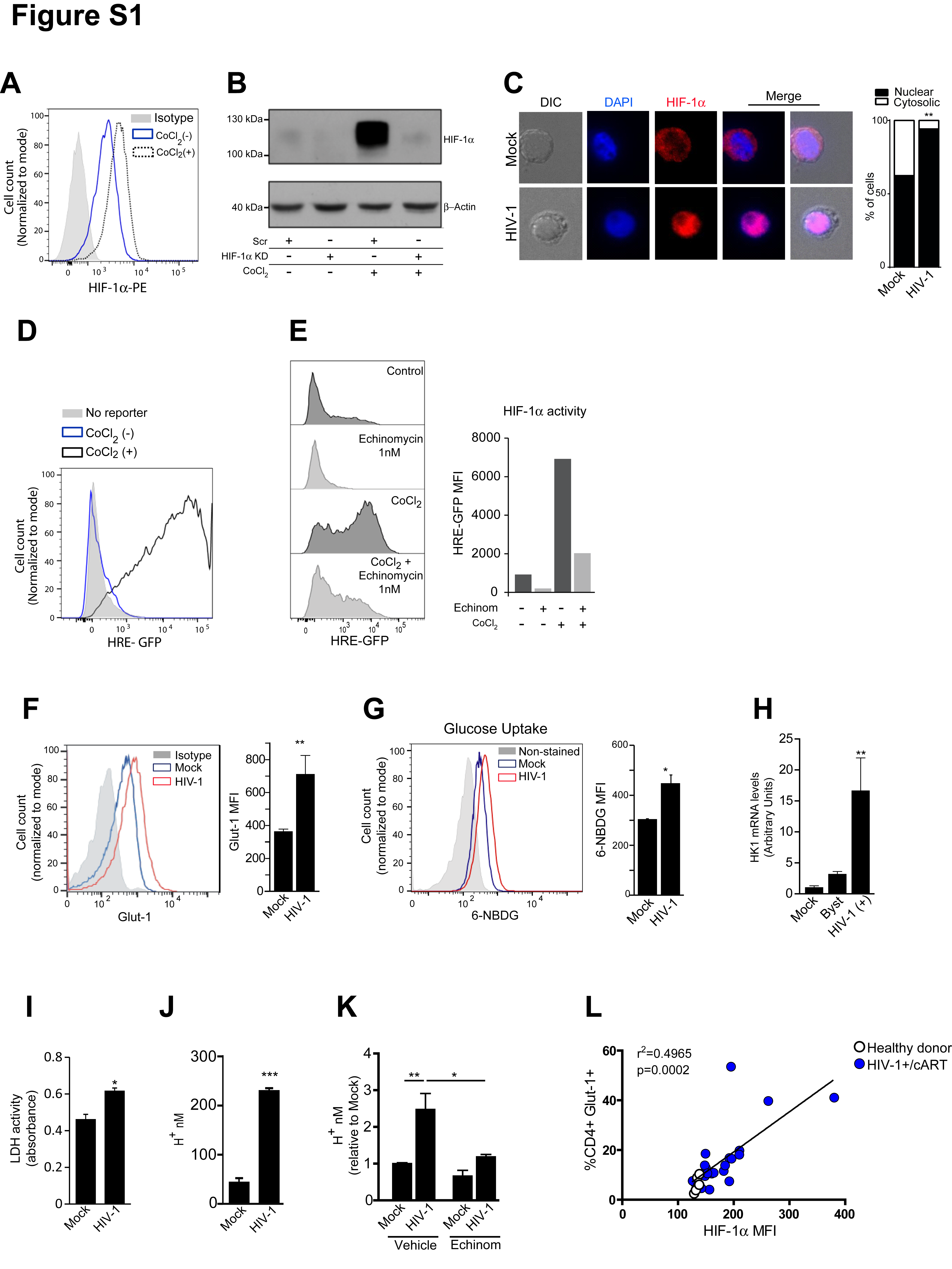

Supplement: FIG S1 [file mbo004184046sf1.tif]

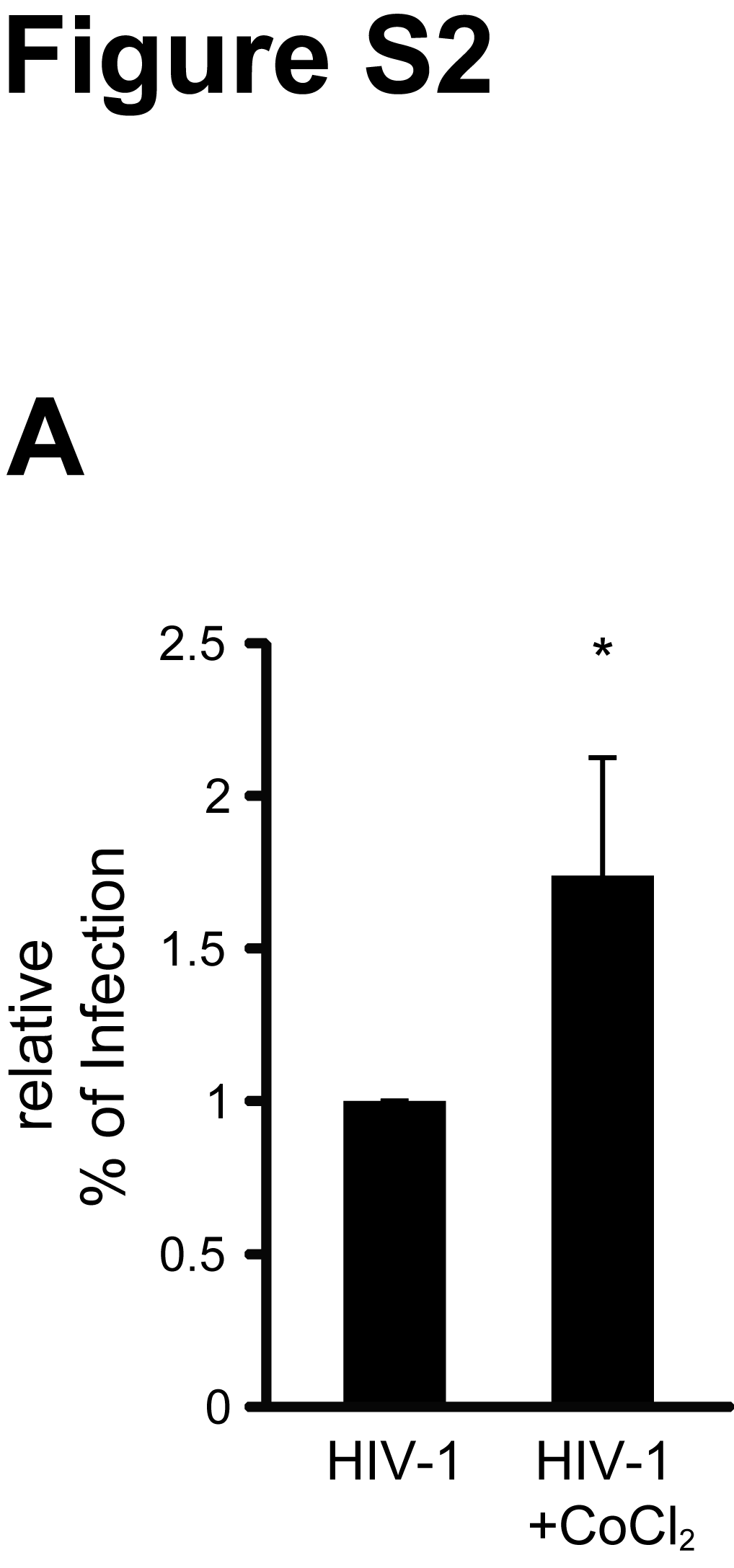

Supplement: FIG S2 [file mbo004184046sf2.tif]

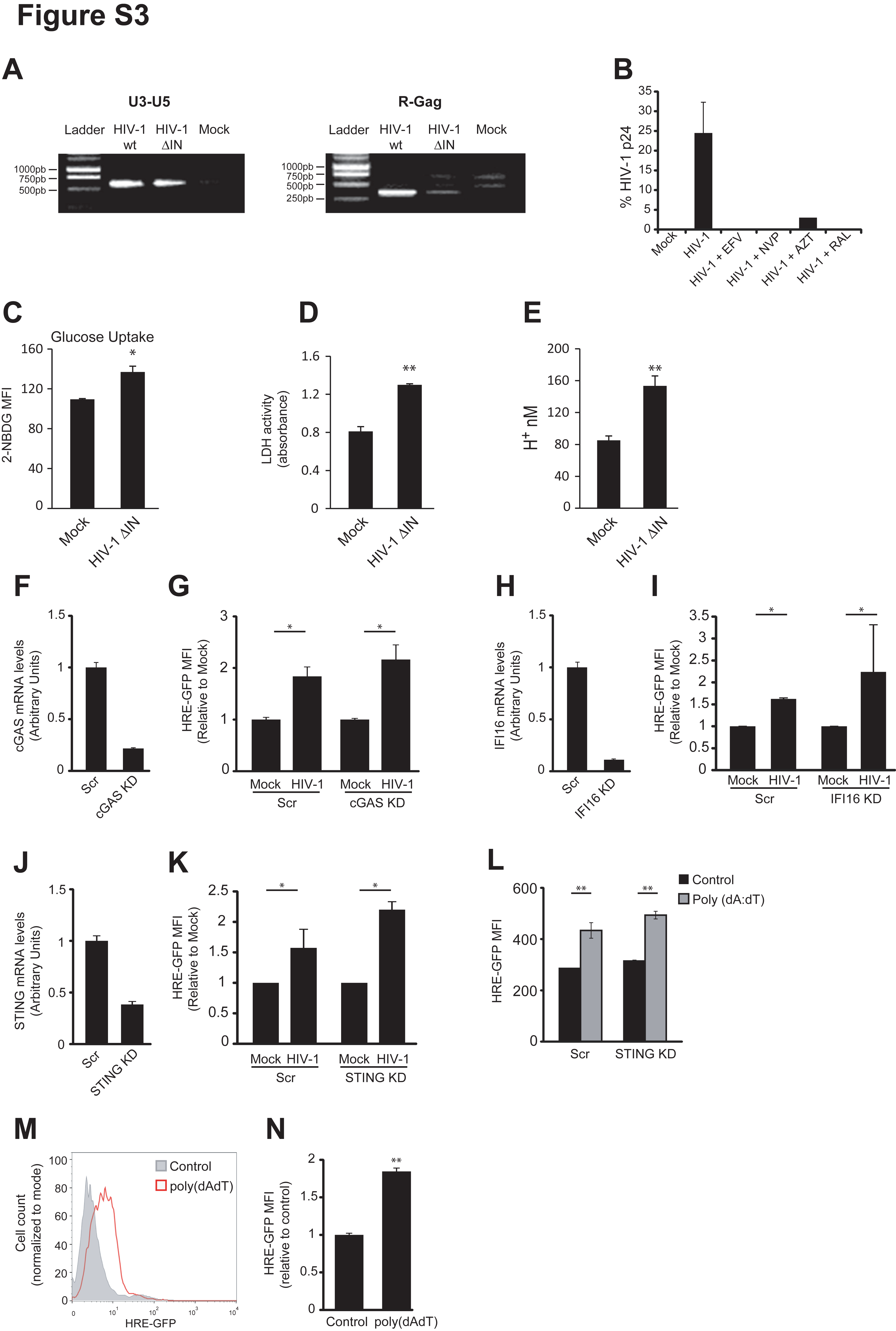

Supplement: FIG S3 [file mbo004184046sf3.tif]

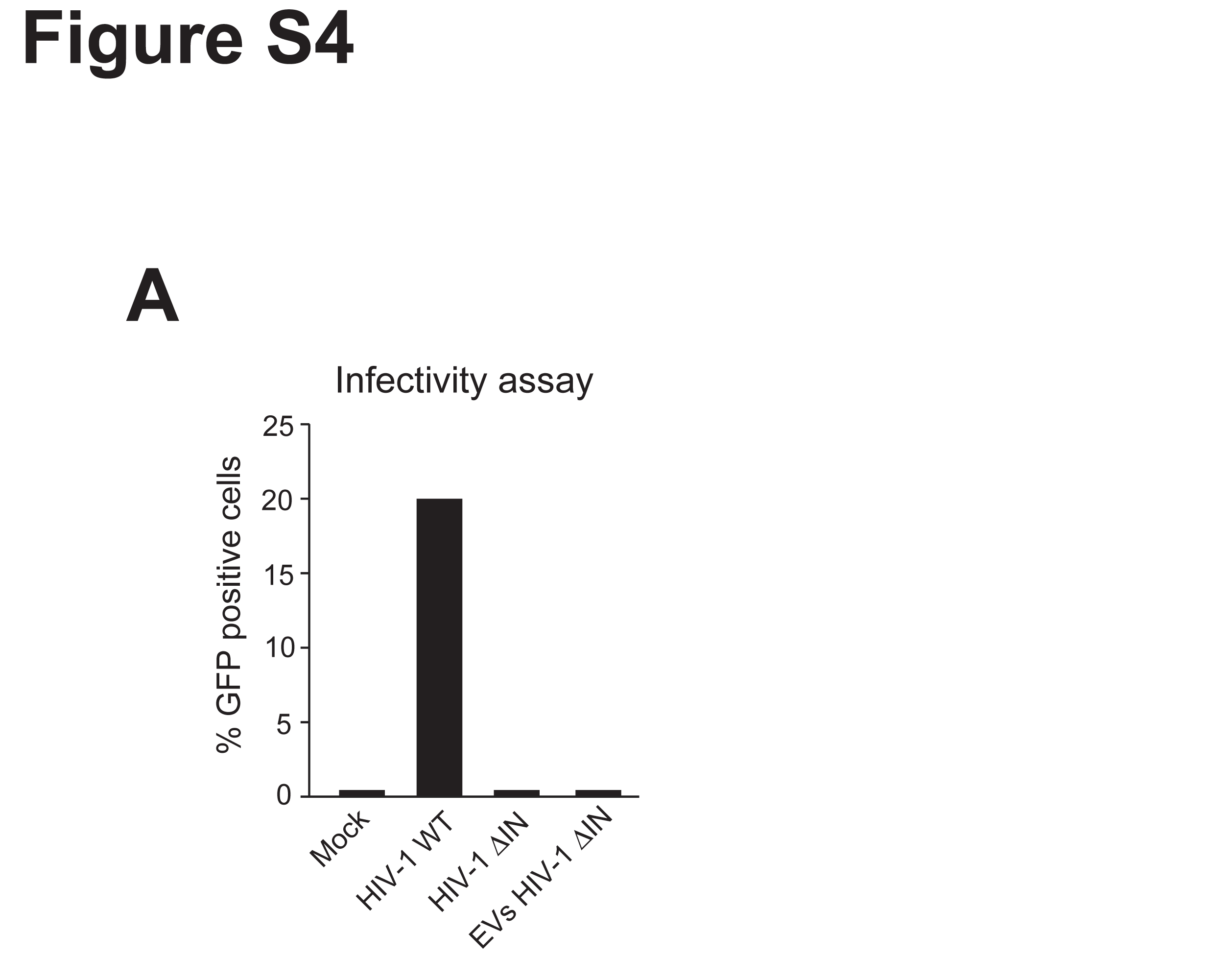

Supplement: FIG S4 [file mbo004184046sf4.tif]

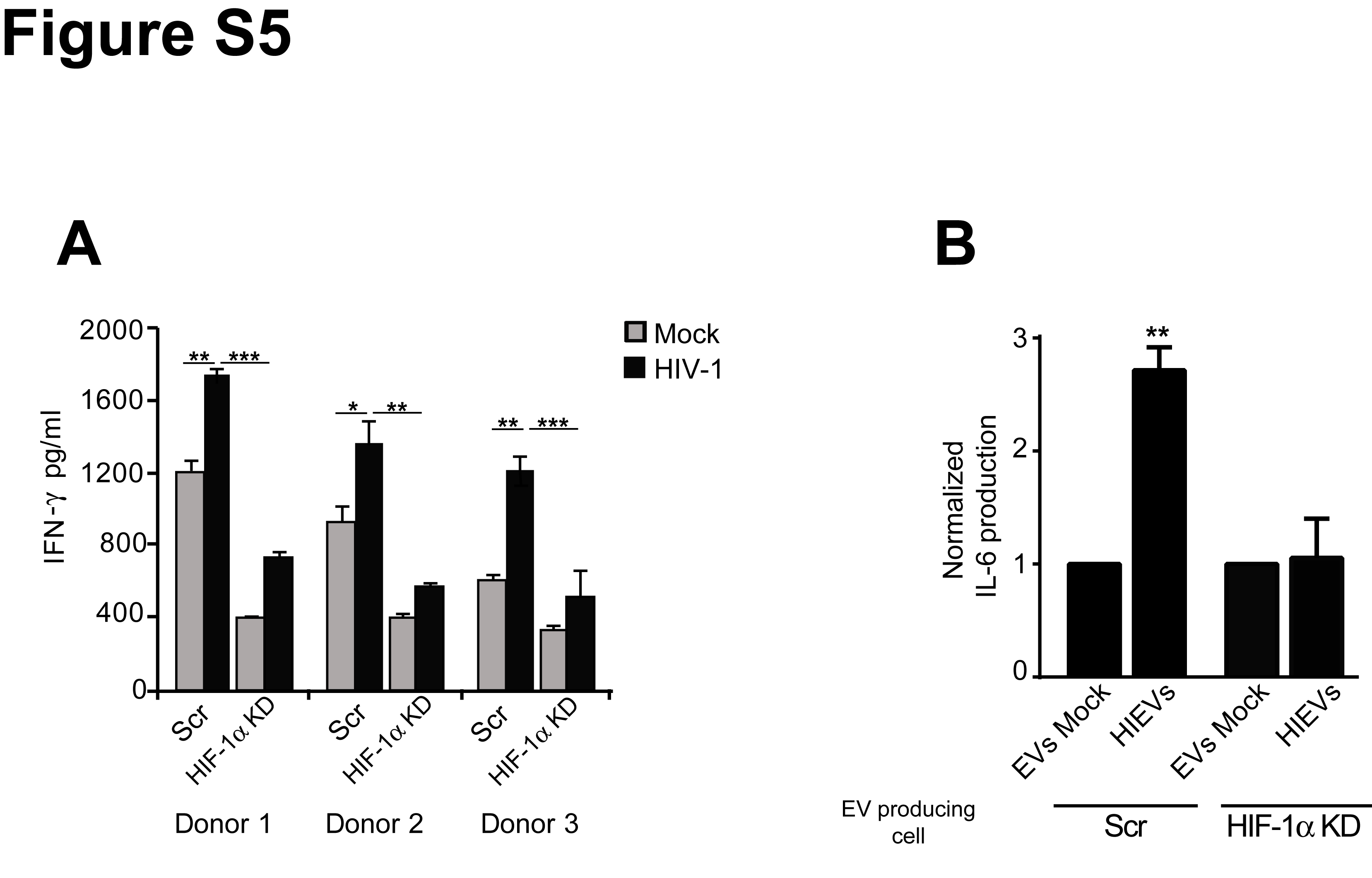

Supplement: FIG S5 [file mbo004184046sf5.tif]

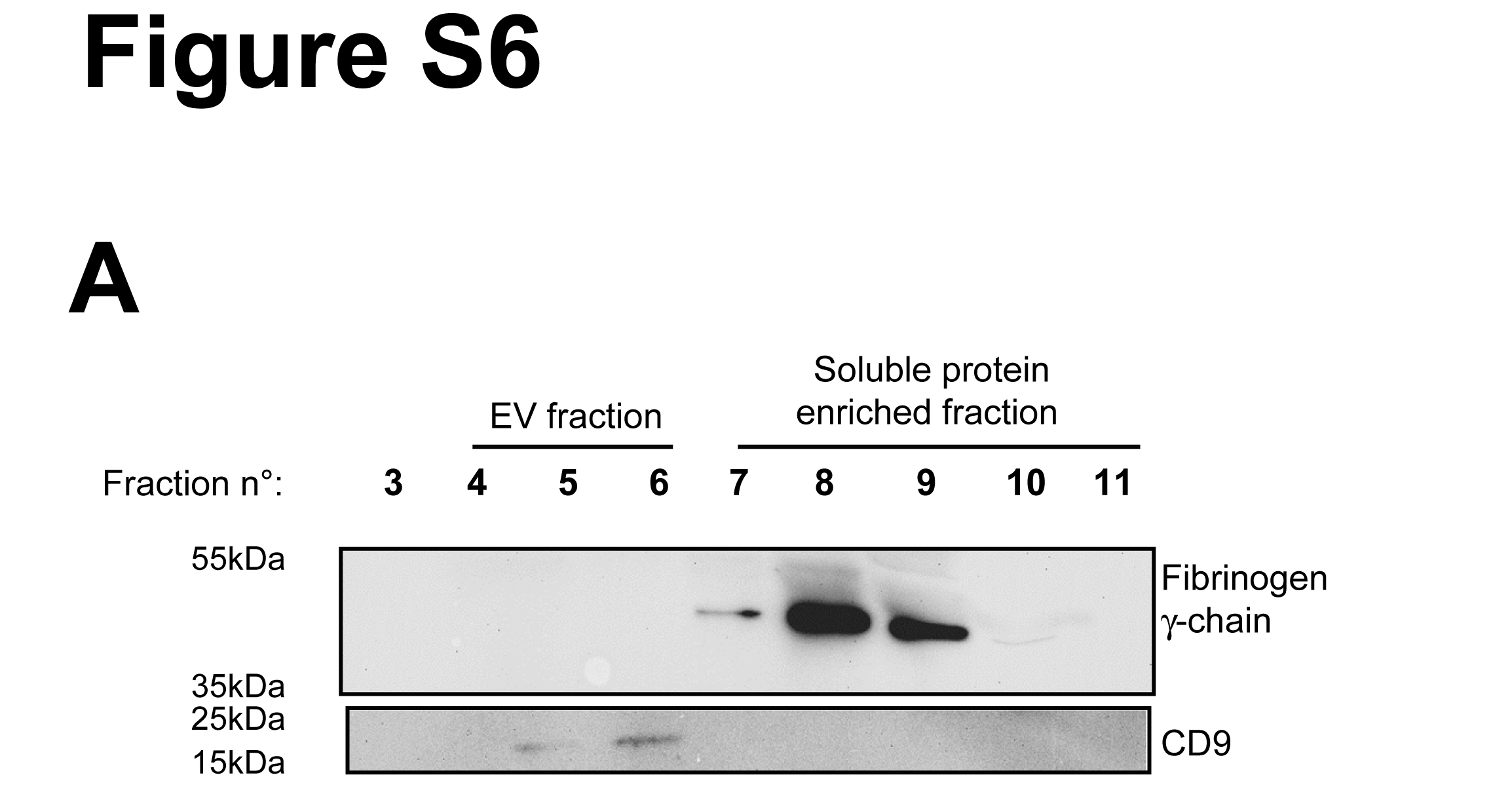

Supplement: FIG S6 [file mbo004184046sf6.tif]
