# Supplementary material for: Induction of HIF-1α by HIV-1 Infection in CD4+ T Cells Promotes Viral Replication and Drives Extracellular Vesicle-Mediated Inflammation
Source: mBio. 2018 Sep 11;9(5):e00757-18. doi: 10.1128/mBio.00757-18 (PMC6134101; doi:10.1128/mBio.00757-18)
Supplement: TEXT S1 [file mbo004184046s1.docx]

**Supplementary Materials and Methods**

**Primary CD4+ T cells and Jurkat T cells culture conditions**

CD4 primary T cells were isolated from buffy coats of healthy donors and purified by negative selection using the CD4+ T cell isolation kit (MiltenyiBiotec). Cells were stimulated with anti-CD3/CD28/CD2 beads (MiltenyBiotec) for two days in RPMI 1640 culture medium (Gibco) supplemented with 10% FBS (Gibco) and 10 U/mL rIL-2.

Jurkat cells were cultured in RPMI 1640 supplemented with10% FBS.

Monocytes were isolated from buffy coats of healthy anonymous donors from the Blood Center of the Mendez Hospital in Buenos Aires, Argentina, by Ficoll and Percoll density gradient centrifugation. Isolated monocytes were plated for 2 h without serum and subsequently differentiated into macrophages by culturing in complete RPMI 1640 medium supplemented with 50 ng/ml GM-CSF for 4 d.

To inhibit the viral replication cycle at different steps we used Enfuvirtide (Fuzeon,Roche), Efavirenz (100nM; SIGMA), Nevirapine (1μM), Raltegravir (30μM) and AZT (10μM; SIGMA).

To inhibit HIF-1α activity, cells were cultured with Echinomycin (1nM, Cayman Chemical).

To inhibit mitochondrial ROS, cells were cultured with MitoTEMPO (500μM, SIGMA)

Transwell experiments were performed with 0.45μM pore membranes (JetBiofil). Mock or ΔIN-HIV-1 infected primary CD4+T cells (5x10^5^ cells/ml) placed in the top chamber were cocultured with Jurkat HRE-GFP cells (1.5x10^5^ cells/ml) placed in the bottom chamber.

To inhibit HIF-1α degradation and promote its activity, Cobalt Chloride (CoCl_2_100 μM) was added to the cultures at the indicated time points.

**Production of VLPs and lentiviral particles to silence gene expression**

VLPs that carry the RNA derived from pLK0.1 were produced by transfection of HEK293T. Briefly, 2.5×10^3^ HEK293T were seeded on a flat-bottom 96-well plate. 24 h later, cells were transfected with a mix of 100 ng pCMV-dR8.91, 100 ng pLKO.1 coding for the indicated shRNA, and 10 ng pCMV–VSV-G per well, using X-treme GENE HP DNA transfection reagent (Roche), following the manufacturer’s recommendations. To generate “empty” VLPs, cells were transfected with 100 ng pCMV-dR8.91 and 10 ng pCMV–VSV-G per well only. Then, 24 h later, medium was replaced, and supernatants containing lentiviral particles were collected at 48 and 72 h after transfection, pre-cleared by centrifugation, aliquoted, and stored at -80°C.

**Visualization of EVs by SEM analysis**

Purified EVs were fixed with PFA 4%, washed once in 1×PBS, resuspended in distilled water and prepared for SEM visualization as described previously ([1](#_ENREF_1)). Samples were visualized in a FE-SEM (Zeiss, model SUPRA 40).

**Visualization of EVs by TEM analysis**

Purified EVs were negatively stained with Uranyl Acetate 2% as described previously ([2](#_ENREF_2)). Samples were visualized in a TEM Zeiss 109T

**Preparation of virus stocks**

Virus stocks were produced by transfection of the corresponding vector (100 ng/well) in HEK 293T cells (2.5×10^3^ cells per well in 96-well plates) using X-treme gene transfection reagent (Roche). When indicated, pseudotyping was achieved by co-transfecting pVSV-G (10 ng/well). Supernatant was harvested at 48 and 72 h after transfection, cleared by centrifugation at 1500 g for 10 minutes and frozen at -80 ºC.

Alternatively, HIV-1 stocks were generated from infected H9 (MN and RF strains) and PM1 (BaL) T-lymphocytic cell lines, concentrated, and stored at −80 °C.

**Lentiviral transduction and gene silencing of cell lines**

To silence gene expression in Jurkat or HeLa cells, a total of 3x10^4^ cells were transduced with lentiviral vectors by spinoculation (2200 rpm, 90 min, 37ºC) in the presence of 8 μg/ml of polybrene. 48h later, transduced cells were selected by the addition of 3 μg/mL puromycin.

**HIV-1 infection**

Jurkat cells (3×10^4^ /0.1 ml) or purified CD4+ T lymphocytes (1x10^5^/0.1 ml) were either incubated (2h) or spinoculated (2200 rpm, 90 min, 37ºC) with VSV-G–pseudotyped NL4-3-IRES-EGFP (50 ng p24/ml [low MOI] or 200 ng p24/ml [high MOI]) or with the corresponding delta-HIV-1 constructs.

GHOST cells (20,000 cells/well) were incubated for two h with HIV-1 NL4-3 (30ng), HIV-1 ΔIN (30 ng), and EVs purified from 6x10^6^ HIV-1 ΔIN infected CD4+ T cells. After 24 h, cells were harvested, and the percentage of GFP positive cells was analyzed by FACS.

MN and RF viruses were added to the J-HRE reporter cell line at a maximum concentration of 500 ng/mL p24 and at serial 1:2 dilutions. BaL was added at a maximum concentration of 250 ng/mL p24 and serial 1:2 dilutions. Virus and cells were incubated at 37 °C for 4–6 hours. Cells were rinsed several times with PBS and spun twice at 400 × g for 5 minutes each.

**qPCR**

Cellular RNAs were isolated using RNeasyPlus Mini Kit (Quiagen), and 1μg RNA was reverse transcribed with M-MLV Reverse transcription (Invitrogen). Only 1/10th cDNA was used for each PCR reaction, performed with SYBR green (Applied Biosystems) on a real-time thermal cycler (STEP ONE PLUS; Applied Biosystems). Cycle thresholds (Ct’s) were normalized to the Ct of β-Actin.

Primer sequences:

β -Actin Fw: 5´-AGGCATCCTCACCCTGAAGT-3´

β -Actin Rev: 5´-GCGTACAGGGATAGCACAGC-3´

HK-1 Fw: 5´-TTGGCTGGAGATGGAAAATCACAC-3´

HK1 Rev: 5´-CACACGATTTTGTGGCATTGA-3´

HIF-1αFw: 5´-TTACAGCAGCCAGACGATCAT-3´

HIF-1α Rev: 5´-ATTGTCTTTTGCTCCATTCCA-3´

cGAS Fw: 5´- AAGAAGAAACATGGCGGCTAT -3´

cGAS Rev: 5´- CCCAGGTCTTTGCGGTCCC -3´

IFI16 Fw: 5´- GCCCAGTGATAGTGAAGGTAC -3´

IFI16 Rev: 5´- AGAACTGTGTCTGTGTAGCCA -3´

STING Fw: 5´- GTTATCAGGCACCCCACAGT -3´

STING Rev: 5´- GATATCTGCGGCTGATCCTG -3´

**Glucose uptake assay**

The fluorescently-labeled glucose analog, 6-N-(7-nitrobenz- 2-oxa-1, 3-diazol-4-yl) amino)-6 deoxyglucose (6-NBDG) (Invitrogen) or 2-(N-(7-Nitrobenz-2-oxa-1,3-diazol-4-yl)Amino)-2-Deoxyglucose (2-NBDG) (Cayman chemical), were used to measure glucose uptake. CD4+T cells were treated for 30 min with 6-NBDG or 2-NBDG at 37ºC in the dark, washed with 1X PBS, and analyzed within 15 min on a FACSCanto (BD).

Glucose concentration in cell culture medium was measured using the GlucCell™ Glucose Monitoring System according to the manufacturer’s instructions.

**Mitochondrial ROS quantification**

Cells were incubated with MitoSOX (5μM, Thermo) for 10 minutes at 37°C, protected

from light. Prior to the FACS analysis, cells were washed three times with PBS. Cells were acquired on a FACSCanto (BD) and analyzed using FACSDiva software (BD).

**Cytokine quantification in cell supernatants**

Several cytokines (IL-2, IL-10, IL-4, IL-6, IFN-γ, TNF-α, and IL-17A) were quantified simultaneously, in supernatants of cell-culture, by flow cytometry using the human Th1/Th2/Th17 CBA kit (BD Biosciences, San Jose, CA, USA) following the manufacturer’s instructions. Acquisition was performed with a FACSCanto flow cytometer (BD Biosciences, San Jose, CA, USA) and samples were analyzed using the FCAP Array v3.0 software (Soft Flow Inc., Pecs, Hungary).

When indicated, IFN-γ, IL-12, IL-10, IL1β, TNF-α and IL-6 in cell culture supernatant were quantified by ELISA according to the manufacturer’s instructions (BD Biosciences).

**Flow cytometry**

For surface staining cells were washed once in 1×PBS followed by incubation for 30 min at 4°C with the corresponding antibody. Prior to the FACS analysis, cells were washed twice with PBS**.** For intracytoplasmic and nuclear staining of HIF-1α, cells were fixed with PFA 4%, washed and permeabilized with methanol for 5min at RT. Cells were subsequently washed with PBS and incubated with the conjugated antibody for 45 min at RT in PBS-0.5% BSA. Cells were acquired on a FACSCanto (BD) and analyzed using FACSDiva software (BD).

**Fluorescence microscopy**

A total of 10^5^ cells were seeded on poly- L -lysine–coated glass coverslips for 30 min, fixed in 4% paraformaldehyde, quenched with 0.1 M glycine, permeabilized in ice-cooled Methanol for 7 min and incubated with the primary mouse anti-human HIF-1α antibody (BD) for 45 min. After extensive washing, cells were incubated with Alexa Flour 594–labeled donkey anti–mouse secondary antibodies (Jackson ImmunoResearch Laboratories, Inc.). The coverslips mounted with DAPI Fluoromount-G (SouthernBiotech) were examined under a Nikon Elcipse Ti-S L100 fluorescence microscope using a Plan Apochromat 60× 1.42 NA oil immersion objective. Images were analyzed using the NIS-Element software.

**Immunoblotting**

Cells were lysed in precooled radioimmunoprecipitation assay buffer (1% Triton X-100, 0.1% SDS, 50 mMTris, pH 7.5, 150 mMNaCl, and 0.5% sodium deoxycholate), supplemented with a cocktail of antiproteases (Roche), and cleared from nuclei by centrifugation at 15,000 g for 5 min. Equal amounts of protein extracts were separated on 4–12% SDS-PAGE, blotted on Polyvinylidene Fluoride Transfer Membrane (Thermo Fisher Scientific) under non-reducing conditions. Blots were revealed using SuperSignal West Pico Chemiluminescent Substrate (Thermo Fisher Scientific).

**Lactate dehydrogenase activity**

To evaluate LDH activity we used the Pierce LDH Cytotoxicity Assay Kit (Thermo scientific) following the manufacturer´s instructions. Briefly, cells were lyzed and the LDH-dependent reduction of Tetrazolium salt to red Formazan was measured at 490nm using a plate-reader spectrophotometer.

**Extracellular acidification measurement**

After infection, cells were incubated for 48 h to quantify the pH of the supernatant as aproxy indicator of glycolysis. pH was quantified with a microelectrode using a Jenco 6173 pH meter.

**Semiquantitative detection of viral DNA**

The PCR technique used to monitor the synthesis of viral DNA in infected cells of each virus was determined 8 h after infection. DNA was extracted using the High Pure PCR Template Preparation Kit (Roche) following the manufacturer’s instructions. The DNA samples were subjected to 30 rounds of PCR amplification with primers designed to amplify intermediate (U3-U5 [sense nucleotides 8687 to 8709, 5′-ACACACAAGGCTACTTCCGTGA-3′; antisense nucleotides 181 to 157, 5′-CTGCTAGAGATTTTCCACACTGAC-3′]), and late (R-gag [sense nucleotides 1 to 22, 5′-GGTCTCTCTGGTTAGACCAGA-3′; antisense nucleotides 355 to 334, 5′-ATACTGACGCTCTCGCACCCAT-3′]) products of reverse transcription. The PCR products were separated on 1% agarose gel and visualized by ethidium bromide staining.

**Statistical analyses**

Data were analyzed using Prism (GraphPad Software). Normality of the data was tested using the Kolmogorov–Smirnov test. Based on the normality test, either one-way ANOVA followed by the Tukey's HSD post test or Kruskal–Wallis followed by Dunn’s post test were used for multiple comparison analyses.

Due to disparity in the FACS values obtained in different experiments (many of them performed in different laboratories), when indicated, data were normalized to control conditions to show pooled results from several experiments.

**Ethics Statement**

Blood samples from HIV-1+ individuals were collected in Melbourne [approval for this study was obtained from the Alfred Health Human Research Ethics Committee (Project 168-08)], and in Buenos Aires [approval for the study was obtained for the Bioethics Committee at Fundacion Huesped (project FH-29)]. In both cases, written informed consent was obtained from each participant.

For in vitro experiments, CD4 primary T cells were isolated from buffy coats of healthy anonymous donors from the Blood Center of the Mendez Hospital in Buenos Aires, Argentina. All donors were > 18 years. In this case, consent was not obtained (Exemption 45 CFR 46.101(b), HHS) because samples have not been collected specifically for this research study and were supplied without personal identifiable information. None of the investigators on this research project have any ready means to link the materials back to living individuals.

1. **Wu Y, Deng W, Klinke DJ, 2nd.** 2015. Exosomes: improved methods to characterize their morphology, RNA content, and surface protein biomarkers. The Analyst **140:**6631-6642.

2. **Raposo G, Nijman HW, Stoorvogel W, Liejendekker R, Harding CV, Melief CJ, Geuze HJ.** 1996. B lymphocytes secrete antigen-presenting vesicles. The Journal of experimental medicine **183:**1161-1172.
